# Supplementary material for: Evaluating the climate impact of aviation emission scenarios towards the Paris agreement including COVID-19 effects
Source: Nat Commun. 2021 Jun 22;12:3841. doi: 10.1038/s41467-021-24091-y (PMC8219675; doi:10.1038/s41467-021-24091-y)
Supplement: Supplementary file 1 — Supplementary Information [file 41467_2021_24091_MOESM1_ESM.pdf]

Development of scenarios

This supplementary material contains two parts:

Part 1: Definition of aviation top-down scenarios
Part 2: Development of ECATS technology scenarios

Part 1: Definition of aviation top-down scenarios

1) Abstract 5

2) Storylines 5

3) Common underlying assumptions 5

4) The WeCare scenario as a basis for the scenario development 7

Model description 7

WeCare Scenario description 8

Expansion of the WeCare scenario to annual values 8

5) Current Technology Scenario (CurTec) 11

Method for Revenue Passenger Kilometre (RPK) 11

Method for Flown-Distances 12

Method for Fuel Use 12

Method for CO_2 Emission 12

Method for NO_x Emission 12

Method for SAF, CO_2-trading, and soot particle number reduction 13

6) Business-As-Usual Scenario (BAU) 13

Method for Revenue Passenger Kilometre (RPK) and Flown-Distances 13

Method for Fuel Use 13

Method for CO_2 and NO_x Emission 13

Method for SAF, CO_2-trading, and soot particle number reduction 13

7) Carbon Offsetting and Reduction Scheme (CORSIA) 13

Method for Revenue Passenger Kilometre (RPK), Flown-Distances, and NO_x Emission 13

Method for SAF 13

Method for CO_2 Emission 14

Method for CO_2-trading 16

Method for soot particle number reduction 16

8) Flightpath 2050 (FP2050 and FP2050-cont) 17

Interpretation of top-level goals for CO_2 and NO_x emissions 17

Method for Fuel use and CO_2-Emissions 17

Method for NO_x-Emissions 17

9) COVID-19 Scenarios 18

Part 2: Development of ECATS technology scenarios

10) Abstract **19**

11) Underlying assumptions **20**

12) Reference aircraft **21**

13) Technologies for future generation aircraft programmes **21**

Fuel consumption reduction potential *22*

NO_x emissions reduction potential *34*

Future low NOx technology *35*

Mission NO_x emission estimates *36*

14) Aviation growth **37**

15) Fleet diffusion **38**

16) Emission distribution from 2015 to 2070 ..... **39**

17) Supplementary References **40**

Part 1: Definition of aviation top-down scenarios

Supplementary material to

Evaluating the climate impact of aviation emission scenarios towards the Paris Agreement including COVID-19 effects

Volker Grewe[1,2,3], Arvind Gangoli Rao[2,3], Tomas Grönstedt[4,3], Carlos Xisto[4,3], Florian Linke[5,3], Joris Melkert[2,3], Jan Middel[6,3], Barbara Ohlenforst[6,3], Simon Blakey[7,8,3], Simon Christie[9,3], Sigrun Matthes[1,3], Katrin Dahlmann[1,3]

[1]Deutsches Zentrum für Luft- und Raumfahrt, Institut für Physik der Atmosphäre, Oberpfaffenhofen, Germany
[2]Delft University of Technology, Faculty of Aerospace Engineering, Delft, Netherlands
[3]ECATS International Association, Brussels, Belgium
[4]Chalmers University of Technology, Mechanics and Maritime Sciences, Gothenburg, Sweden
[5]Deutsches Zentrum für Luft- und Raumfahrt, Lufttransportsysteme, DLR-Hamburg, Germany
[6]Royal Netherlands Aerospace Centre (NLR), Amsterdam, Netherlands
[7]University of Birmingham, Mechanical Engineering, Birmingham, United Kingdom
[8]University of Sheffield, Mechanical Engineering, Low Carbon Combustion Centre, United Kingdom
[9]Manchester Metropolitan University, Department of Natural Sciences, Manchester, United Kingdom

1) Abstract

In order to estimate the future climate impact of aviation and especially to relate this to the Paris Agreement, a description of future pathways for aviation is necessary. In this supplementary material we explain in detail how we derive and analyse the top-down scenarios. Eight scenarios are taken into account. Five scenarios are addressing top-level targets which all have the same projection of transport volume (in passenger kilometres), but differ in the air traffic system efficiency, technological efficiency, and political measures. Three scenarios are dedicated to the developments due to COVID-19. In all scenarios, we combine top-level assumptions with a more detailed description of the air transport system. Latter is based on results from the DLR-Project WeCare[1]. The scenarios are implemented in an EXCEL-file. We first describe the storylines for the five scenarios, those scenario assumptions, which are common to all scenarios, the WeCare base traffic sample and evolution, and then more specifically describe the individual scenarios. The structure of this scenario definition is can also be found in the EXCEL-file.

2) Storylines

We define five scenarios, which are described in Supplementary Table 1, and which are also available in the 'Storyline' sheet of the scenario EXCEL-file:

Supplementary Table 1: Description of top-down scenarios.

Short Name	Long Name	Description
**CurTec**	Current Technology	Current (2012) technology is used as-is and no further political measures are implemented<br>= "What happens if nothing happens"<br>= "NoAction"
**BAU**	Business as usual	"Business as usual" increase in fuel efficiency without any specific aims to reduce the climate impact of aviation
**CORSIA**	Carbon-Offsetting Scheme[2]	As BAU, with a carbon neutral growth from 2020 onwards
**FP2050**	Flight-Path 2050	As BAU, but including technology advancements, which are introduced according to Flightpath 2050
**FP2050-cont**	Flight-Path 2050, continuous implementation	As FP2050, but technology advancements are introduced earlier and a smooth transition is realised

3) Common underlying assumptions

All scenarios have the identical evolution in transport volume, defined by the revenue passenger kilometres, which resemble ICAO data for the past (1971-2017). The data is extrapolated to the past and future with the assumption of a slow decrease in traffic growth rates in the future (for details see Section "Current Technology Scenario") taking the results from the WeCare project into account[1].

---

[1]WeCare: "Utilizing **We**ather information for **Cl**im**a**te efficient and eco efficient futu**re** aviation"; see Grewe et al.[1].

[2]CORSIA: "Carbon Offsetting and Reduction Scheme for international Aviation"; International Civil Aviation Organisation (ICAO), see e.g. www.icao.org

The geographical distribution follows the emission inventories developed within the WeCare project. Two time horizons are taken, one for the past (=2012) and one representative for the future (2050). Total amounts of emissions are scaled according to the individual scenarios.

All scenarios are identical between 1940 and 2018, and deviate afterwards, according to the scenario assumptions. For describing the past evolution of aviation, ICAO and IATA data are used (Supplementary Figures 1-3).

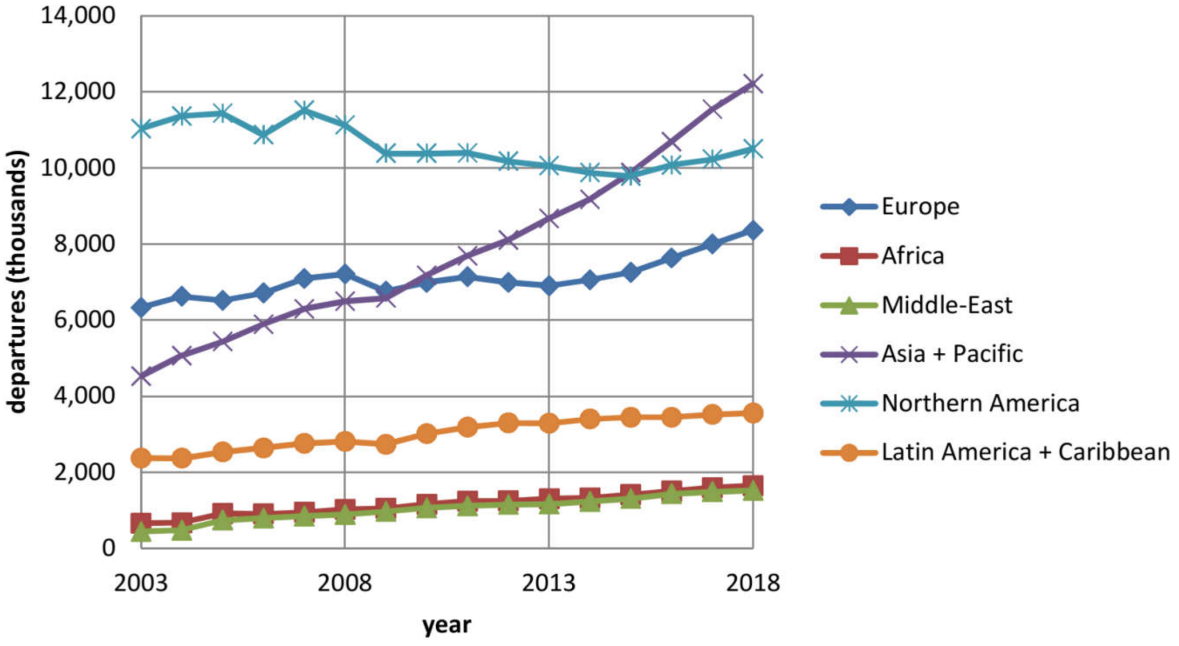

Supplementary Figure 1: Trends in aircraft departures (flights) per region (data from ICAO annual reports).

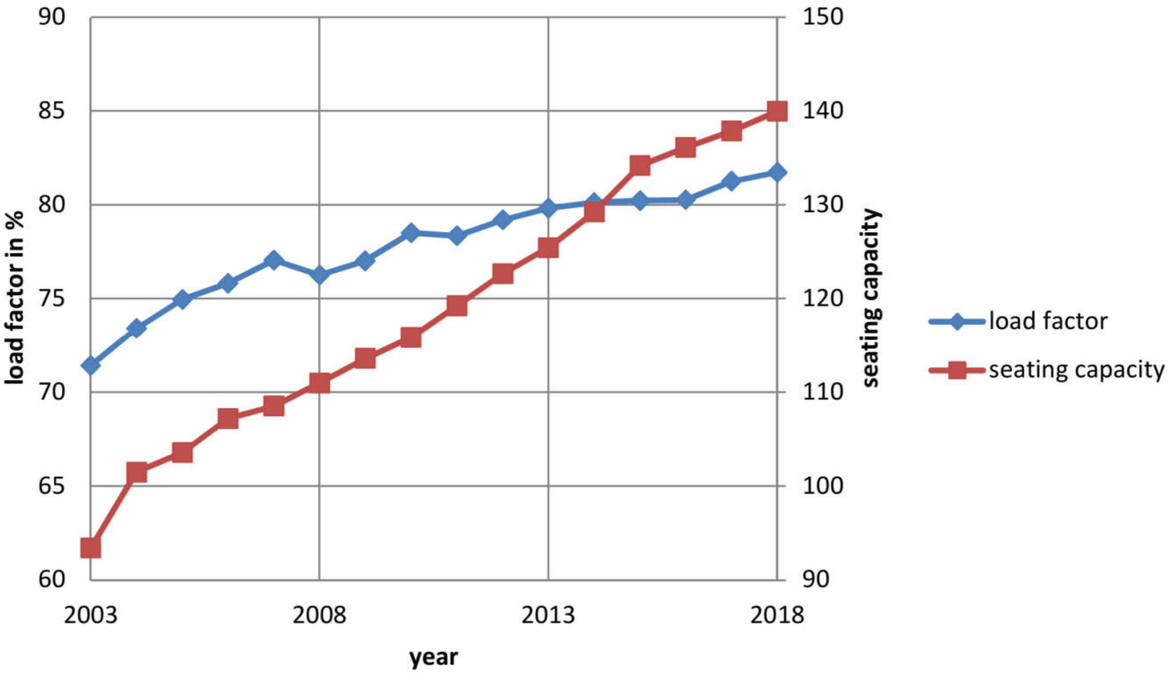

Supplementary Figure 2: Trends in aircraft seating capacity and load factor (data from ICAO annual reports).

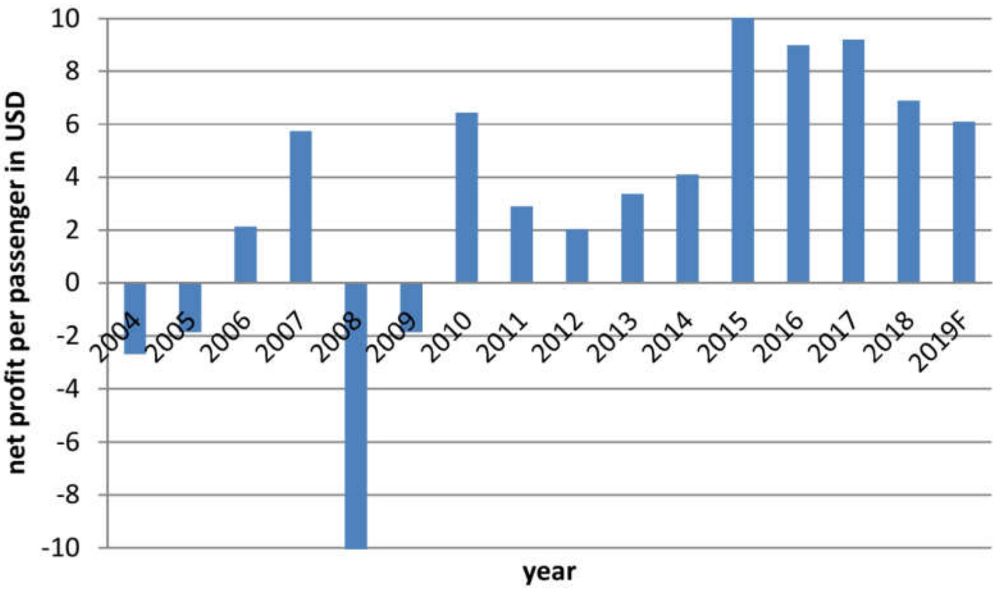

Supplementary Figure 3: Trends in net profit per passenger (data from IATA Airline Industry Economic Performance)

4) The WeCare scenario as a basis for the scenario development

The scenario development between the time period of the ICAO data set and the year 2050 is based on a modelling approach developed within the DLR-project WeCare. Here, we first describe briefly the modelling framework and then summarise the results. The individual modelling parts, as well as the whole modelling framework and respective results are published elsewhere (see below). Here we give a summary, only.

Model description

An overview of the modular assessment framework developed within the DLR-project WeCare and is summarised in project overview in Grewe et al.[1] and the summary presented here is based thereon. The assessment framework is based on a 4-layer philosophy for a generic description of the future passenger air traffic in networks on a global scale. The four layers comprise: (1) the origin-destination passenger demand network; (2) the passenger routes network; (3) the aircraft movements network, and (4) the trajectories network[2]. The approach is implemented in the model chain called AIRCAST[3] (air travel forecast). Due to the global network layer modelling architecture on a city pair level, information on how many passengers will travel between which city-pairs in a given future year, which routes will be chosen by the passengers as well as how many aircraft and which size of aircraft will be operated on each flight segment worldwide can be provided in terms of quantitative scenarios. As a starting point for the derivation of air passenger demand between settlements exogenous socio-economic scenarios are initialized[4,5]. As inputs, the forecast published by Randers[6] and the five scenarios of the International Futures Global Modeling System[7] (IFs) are adopted. Passenger route probabilities are calculated based on historical ADI (Sabre Market Intelligence, formerly Airport Data Intelligence) data. The passenger routes network consists of two sublayers: (2a) the passenger route network and (2b) the passenger segment network. The subsequent (3)

aircraft movements network with aircraft sizes and frequencies is calculated applying the DLR frequency-capacity-model FoAM[8] (Forecast of Aircraft Movements) and fleet renewal model FFWD[9] (Fast Forward). It also consists of two sublayers: (3a) the aircraft movements network by seat categories and (3b) the aircraft movements network by aircraft type and aircraft generation. Modelling the structural evolution of global air passenger flows and aircraft movements over time is a necessary means to quantify future shifts induced by heterogeneous growth in world regions and airline as well as passenger behaviour[10]. Finally, using these aircraft movements, the amount, locus and time of emissions can be computed by trajectory simulations under realistic operational conditions with GRIDLAB[11] (Global Air Traffic Emissions Distribution Laboratory). The tool translates aircraft movements into 4D aircraft trajectories based on aircraft performance data from EUROCONTROL (Base of Aircraft Data, BADA 4) and simplified flight mechanics. Engine emissions are determined by applying the Boeing Fuel Flow Method 2[12] in combination with engine ground test data from the ICAO Engine Emission Databank[13].

WeCare Scenario description

The WeCare scenario gives detailed information on transport volumes and emissions for 2012, 2015, and then every 5 years until 2050. A main characterization of the data set can be found Supplementary Table 2.

Supplementary Table 2: Temporal evolution of the WeCare scenario

Time	Flights (Number)	Distance (km)	Fuel (kg)	NO_x Emission (kg(NO_2)/year)
2012	3.08E+07	5.33E+10	2.46E+11	3.51E+09
2015	3.40E+07	5.99E+10	2.78E+11	3.97E+09
2020	3.90E+07	7.04E+10	3.30E+11	4.72E+09
2025	4.51E+07	8.27E+10	3.92E+11	5.61E+09
2030	5.15E+07	9.55E+10	4.57E+11	6.54E+09
2035	5.78E+07	1.09E+11	5.28E+11	7.57E+09
2040	6.39E+07	1.22E+11	5.97E+11	8.56E+09
2045	6.95E+07	1.34E+11	6.62E+11	9.49E+09
2050	7.39E+07	1.43E+11	7.16E+11	1.03E+10

Expansion of the WeCare scenario to annual values

We are using the information on growth rates from the WeCare scenario for the period 2012 to 2050 to define in more detail annual changes in transport volume, flown kilometres and emissions to expand that to annual changes, which are used in the end for the scenario definition. Hence, this is an interim step. How it eventually is used is described in the Section "Current Technology Scenario". The parameters for this interpolation method are shown in Supplementary Table 3 and it is comprised of 6 steps, shown in Supplementary Table 4.

Supplementary Table 3: Variables used in the interpolation method.

Variable	Description	Unit
i=1,…,*n*	n is the number of available time steps in the WeCare data set (=9)	-
x(i)	Year of time step i, e.g. *x(1)*=2012, …	-
y(i)	respective value of the time series, e.g. RPK	e.g. pax-km
K	years for final annual change rates	-
γ	Mean annual change between *x(i-1)* and *x(i)* as calculated in step 1	-
β	Annual changes based on nodes in step 3	-
α	Correction factor calculated in step 4	-

Supplementary Table 4: Six steps of the used interpolation method.

Step	Description
**1. exponential increase rates**	Calculate exponential increase γ between time steps *x(i)* and *x(i+1)*
**2. Define nodes and their increase rates**	Define nodes: *x(i-1)*, *x(i)* and x(i+1) are given; then the annual increase rates of the node can be interpolated linearly *y(i)=y(i-1)+(x(i)-x(i-1))*(y(i+1)-y(i-1))/(x(i+1)-x(i-1))*.
**3. Estimate time series on annual basis**	Calculate time series of annual changes β on interpolated nodes
**4. Correction factor**	Calculate correction factor to match original values
**5 Final annual changes**	Calculate annual changes based on the corrected annual changes
**6. Final check**	Compare mean annual changes of original WeCare data with results from step 5.

The values of $\alpha, \beta, and\ \gamma$ are calculated as follows:

$$\gamma(k) = \left(\frac{y(i+1)}{y(i)}\right)^{\frac{1}{x(i+1)-x(i)}} - 1 \qquad \text{for years } k \text{ with } x(i) \le k \le x(i+1)$$

$$\beta(k) = \gamma(k) + \left[\gamma\big(x(i+1)\big) - \gamma\big(x(i)\big)\right]\frac{k - x(i)}{x(i+1) - x(i)} \text{ for years } k \text{ with } x(i) \le k \le x(i+1)$$

$$\alpha = \left(\tfrac{y(i+1)}{y(i)}\right)^{1/(x(i+1)-x(i))} \left[\textstyle\prod_{j=x(i)}^{x(i+1)}(1+\beta(j))\right]^{-1/(x(i+1)-x(i))} - 1,$$

$$\alpha = \gamma(k)\left[\prod_{j=x(i)}^{x(i+1)}(1+\beta(j))\right]^{-1/(x(i+1)-x(i))} - 1$$

An aircraft age analysis was conducted using data from FlightGlobal (now: Cirium) Flight Fleets Analyser. A complete list of registered aircraft that were in service in July 2018 was exported from the database. For each individual aircraft, besides model name, engine type, operator name and registration number, also the age of the aircraft were included. All aircraft were categorized according to their size and the average fleet age of all single-aisle (=narrow-body) and twin-aisle

aircraft (=wide-body) in service in July 2018 was computed to 10.9 years. Supplementary Figure 4 shows histograms of the worldwide single-aisle fleet as of 23 July, 2018, and twin-aisle fleet respectively.

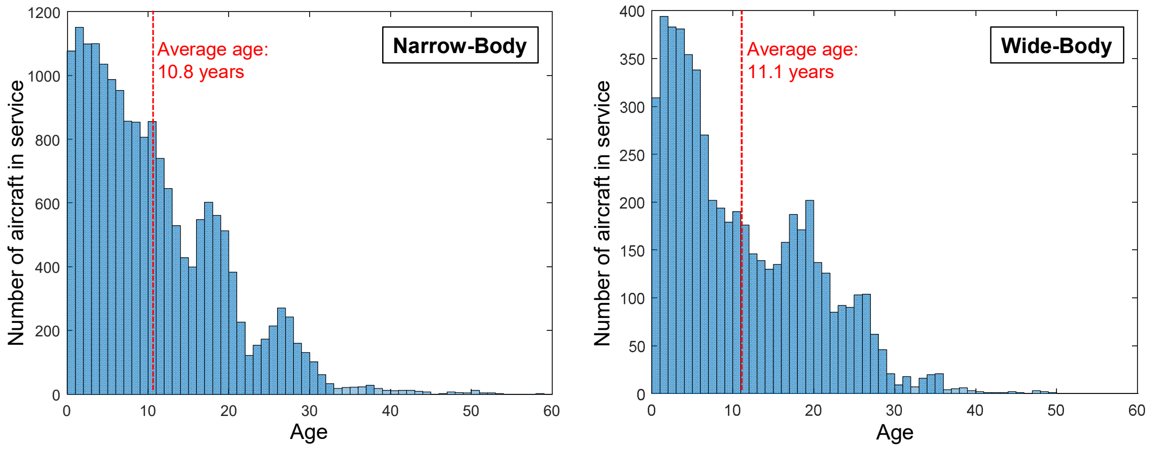

Supplementary Figure 4: Age distribution of single-aisle and twin-aircraft in-service 2018. Mean age is indicated with a red line.

Cooper et al.[14] provided a forecast of the global in-service aircraft fleet and estimated an average fleet age of 9.7 years in the year 2027. Based on the trend that can be observed by comparing the age data of the current fleet with the forecast, we assumed an average fleet age of 11 years for 2012 and 9 years for 2050. This is a relevant number for estimating the penetration of new technologies to achieve the Flightpath 2050 goals.

5) Current Technology Scenario (CurTec)

In the following we address the methodology for calculating the temporal developments of various parameters, which are given in Supplementary Table 5 and includes the use of sustainable alternative fuels (SAF).

Supplementary Table 5: Overview on used parameters and units

Annual values	Description	Unit
RPK	Revenue passenger kilometre	pax-km
Dist	Flown aircraft distances	km
Fuel_Cons	Fuel consumption	kg/km
Fuel_Eff	Fuel Efficiency = 1/*Fuel_Cons*	km/kg
Fuel	Total fuel use (including SAF[3])	kg
SAF_tot	Total SAF use	kg
SAF_contr	Contribution of SAF for the fuel amount above the 2020 value	%
SAF_CO2_red	Net CO_2 reduction for the use of SAF	%
SAF_red	Reduction in CO_2 emission due to the use of SAF	kg
CO2	Carbon dioxide emission	kg
EICO2	Emission index of CO_2 (constant)	kg/kg
EICO2-SAF	Factor to be multiplied to EICO2 to obtain reduced emission of SAF	-
NOx	Nitrogen oxide emission	kg
EINOx	Emission index of nitrogen oxide emission	kg(NO_2)/kg
SAF	SAF contribution	%
Trade	Part of the emissions, which are traded	%
Part	Changes in soot number concentration emissions wrt today	%

Note that the RPK calculation is for every top-down scenario identical.

Method for Revenue Passenger Kilometre (RPK)

Data from ICAO annual reports[4] for the RPK is available for the time period 1971 to 2017. We analyse the annual increase rates and estimate from that past and future rate changes. The actual RPKs are then calculated based on the estimated rate changes:

$$RPK(k+1) = RPK(k) \times rate\_RPK(k+1) \text{ and}$$

$$RPK(k) = \frac{RPK(k+1)}{rate\_RPK(k+1)}, \text{respectively}.$$

We found a mean increase rate of 6.22%/year for the period 1971 to 2017. Hence, for the period prior to 1971 we extrapolate backwards with a rate of increase of 6%/year. For 2017 to 2025 we define a linear transition from the ICAO annual changes of 6%/year to the simulated increase rates of the WeCare scenario. After this transition phase, we take the annual rates from the WeCare scenario

[3] Sustainable Alternative Fuels
[4] https://www.icao.int/publications/Pages/annual-reports.aspx

which shows an increase of 1.2%/year in 2050. A further steady decrease of the RPK is assumed for the far future with rates of 1.0%/year and 0.8%/year in 2075 and 2100, respectively.

Method for Flown-Distances

Flown distances are calculated based on the RPKs with estimates of the ratio of RPK to flown distances. We make use of the overlap between the period, where ICAO data are available (1971 to 2017) and where WeCare data are available (2012 to 2050). For the WeCare period, we use the ratio of RPK to flown distances and multiply them with the calculated RPKs. This ratio is approximately 82 pax-km/km in 2050 and we linearly enhance it to 85 pax-km/km in 2100 and linearly reduce it to 40 pax-km/km in 1940 (expert judgement). The Flown Distances (*DIST, km*) is then:

$$Dist(k) = RPK(k) * ratio\_RPK\_FL\_Dist(k).$$

Method for Fuel Use

The fuel use is calculated simply based on the fuel efficiency (km/kg) and the fuel consumption (*fuel_cons* in kg/km) per distance, respectively:

$$Fuel(k) = Dist(k) * fuel\_cons(k).$$

For the period 2012 to 2017 the fuel consumption of 4.61 kg/km is taken from the WeCare data. The value from 2017 (4.67 kg/km) is prescribed for the future to follow the top-level definitions of the current technology scenario. Prior to 2012, the fuel consumption is increased linearly to roughly the double value of 8.0 kg/km in 1940. Note that the assumptions at the beginning and the end of the regarded period, although having an impact on the emission scenarios, their impact on calculated temperature changes is limited, because early emissions are small and late emissions have a negligible impact on temperature till 2100 (only after 2100, which is not regarded in the calculations).

Method for CO_2 Emission

The emissions of CO_2 are derived by multiplying the fuel with the CO_2 emission index of 3.14 kg/kg.

$$CO2(k) = Fuel(k) * EICO2(k).$$

Method for NO_x Emission

The emissions of NO_x are derived by multiplying the fuel with the NO_x emission index:

$$NOx(k) = Fuel(k) * EINOx(k).$$

The emission index of NO_x is taken from the WeCare data for the period 2012 to 2017 and prescribed to the respective 2017 value of 0.0143 kg(NO_2)/kg for the future following the top-level definition of the scenario. For the past, the *EINOx* is increasing to 0.020 kg(NO_2)/kg in 1940.

Method for SAF, CO_2-trading, and soot particle number reduction

According to the scenario definition, all three values (*SAF*, *Trade*, *Part*) are set to 0%.

6) Business-As-Usual Scenario (BAU)

The BAU scenario is largely based on the CurTec scenario but includes an increase in fuel efficiency.

Method for Revenue Passenger Kilometre (RPK) and Flown-Distances

RPKs and flown distances are identical to the CurTec scenario.

Method for Fuel Use

For the time 1940 to 2017 the fuel use is identical to that of the CurTec scenario. For the time 2018 to 2100, the fuel consumption is calculated with a decrease of 1% per year in 2018, which linearly reduces until 2100 to 0.25%/year. The fuel used every year is calculated as in the CurTec scenario.

Method for CO_2 and NO_x Emission

The method is identical to the CurTec scenario. Note that the fuel use differs and hence the CO_2 and NO_x emissions.

Method for SAF, CO_2-trading, and soot particle number reduction

The method and numbers are identical to the CurTec scenario.

7) Carbon Offsetting and Reduction Scheme (CORSIA)

The CORSIA scenario is based on the BAU scenario and differs only in the CO_2 emissions, which are assumed to be constant from 2020 onwards due to both, the use of SAF and emission trading.

Method for Revenue Passenger Kilometre (RPK), Flown-Distances, and NO_x Emission

RPKs and flown distances are identical to the BAU scenario.

Method for SAF

For the period 1940 to 2019, no SAF and no CO_2 trading is taken into account. From 2020 onwards a SAF contribution (*SAF_contr*) is assumed for CO_2 emissions above those in 2020. The introduction of significant volumes of SAF into the market will alter the equations described in the CurTec and BAU scenarios. Rather than considering the fuel consumption per unit distance it is necessary to convert to the energy consumption per unit distance (and in turn the total energy requirement) and then to recalculate the overall fuel consumption of both conventional and SAF using the energy balance below:

$$E_{total} = e_{fossil}M_{total} = e_{fossil}Fuel(k) = e_{fossil}M_{fossil} + e_{SAF}M_{SAF}$$

The actual energy content (MJ/kg) of fuel varies depending on production processes and feedstocks. The average energy content of conventional fossil fuels is 43.24 MJ/kg[15] as can be seen in Supplementary Figure 5. SAF are typically more severely hydro-processed products as a result of the upgrading steps required to meet the requirements of ASTM D7566 and are consequently of a higher energy content than fossil fuels. For this study, we will take an average energy content of the neat SAF to be 44.3 MJ/kg. This typically would result in a fuel which either at or beyond the lowest end of the density specification for aviation fuels (775 kg/m^3). For this study the SAF will be assumed to be blended with conventional fuels for the majority of scenarios such that the blended product would be compliant with the gas turbine aviation fuel specifications, namely ASTM D1655 and DEFSTAN 91-091.

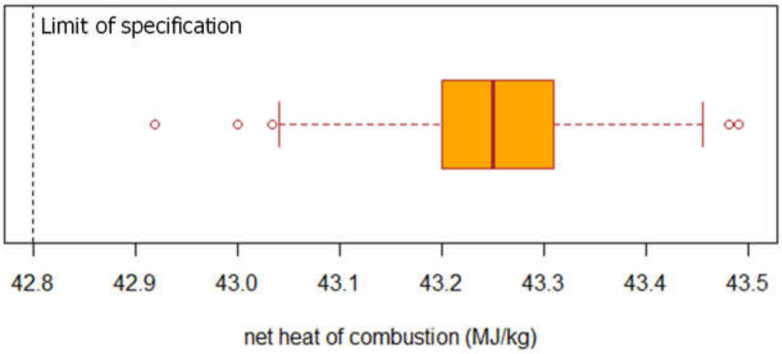

Supplementary Figure 5: Box plot of conventional fossil aviation fuel energy content[15]. The vertical lines refer to 1%, 5%, 50% (median), 95%, and 99%; the circles indicate the outliers.

The fuel equation can therefore be rewritten as:

$$Fuel(k) = M_{fossil} + \frac{e_{SAF}}{e_{fossil}} M_{SAF}$$

Where $\frac{e_{SAF}}{e_{fossil}} = 1.025$, reflecting the higher energy content of the SAF. As a result, a third of the fuel is SAF in 2100.

Method for CO_2 Emission

For the case of purely fossil fuel-based flight, the emissions of CO_2 are derived by multiplying the fuel with the CO_2 emission index (EI_{CO2}) of 3.14 kg/kg.

$$CO2(k) = Fuel(k) * EICO2(k).$$

Blakey et al.[16] showed that the use of SAF (here: synthetic paraffinic kerosene, SPK) leads to a decrease in fuel consumption (2.5%, their tables 3 and 4 for SPK at spec limit) due to a higher energy density, which decreases the specific fuel flow at cruise. Additionally, the emission index for CO_2 is reduced, which in total leads to a reduction in CO_2 emission of roughly 3%. Similar results were found on test flights with LH and KLM, leading to a 1% to 2% CO_2 reduction for a 50-50 blend[5]. Hence, we are taking the different EICO2 and different lower heat value of SAF compared to purely fossil fuels into account:

The CO_2 emission index can be derived from the Hydrogen and Carbon content of the fuel (Supplementary Figure 6). Although this measurement is not required in the fuel specifications or usually reported, some datasets do contain measurements which can provide validation for this *EICO2*. This data suggests an average of 3.16 kg/kg is a more suitable value for *EICO2* from the US market, and that a value of 3.14 kg/kg is representative of a lighter, more hydro-treated fuel.

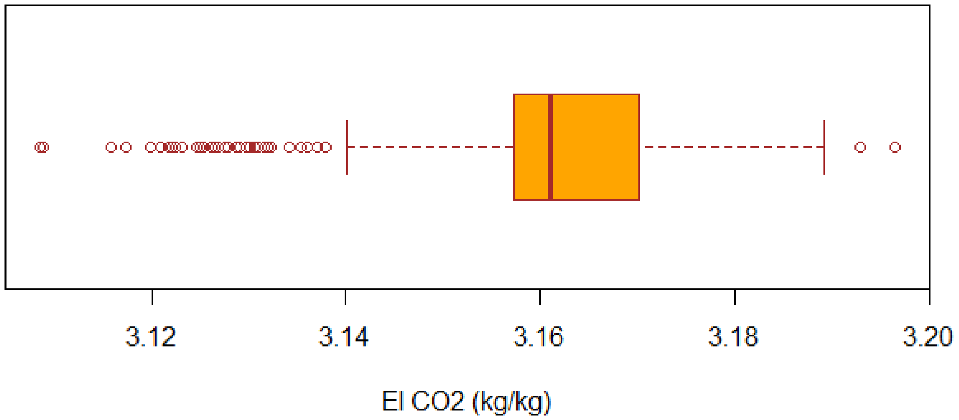

Supplementary Figure 6: Calculated EI_{CO2} for 7,000 fuels in the 2008 PQIS fuels survey[17], based on hydrogen content measurements. The vertical lines refer to 1%, 5%, 50% (Median), 95%, and 99%; the circles indicate the outliers.

Due to the higher hydrogen content of SAF, a lower *EICO2* of around EI_{SAF}=3.10 kg/kg is used in the subsequent calculations. Therefore, the CO_2 emissions equation is altered to the following form:

$$CO2(k) = M_{fossil}EI_{fossil}. + (1-\alpha)M_{SAF}EI_{SAF}$$

where α is the ratio of CO_2 emissions as a result of the production of SAF compared to conventional fuels. This ratio is mandated to be below 65% by 2025 in the EU Renewable Energy Directive II[18]. In this study, we envisage that this ratio will increase over the time period of this study to 80%, which represents the best available technology in 2020.

[5] https://www.greenaironline.com/news.php?viewStory=348

In this study, the total mass of fossil fuel provided for the aviation sector is a variable depending on the energy requirements of the aircraft hardware and the projected distance flown globally (*Fuel(k)*) as well as the availability of SAF (M_{SAF}). Therefore, the above equation can be combined with the fuel use equation to give:

$$CO2(k) = \left(Fuel(k) - \frac{e_{SAF}}{e_{fossil}} M_{SAF}\right) EI_{fossil}. + (1-\alpha) M_{SAF} EI_{SAF}$$

Rearranging:

$$CO2(k) = Fuel(k) EI_{fossil} - M_{SAF}\left(\frac{e_{SAF}}{e_{fossil}} EI_{fossil} - (1-\alpha) EI_{SAF}\right)$$

This equation clearly shows how the use of SAF can reduce the CO_2 emission of fuel by three variables:

- The increased energy content of the fuel (resulting in a lower fuel burn rate)
- The lower carbon content of the fuel (resulting in a lower *EICO2*)
- The lower carbon impact of fuel production (1-α)

Using 3.14 kg/kg this reduces the constant in the equation to in 2020:

$$CO2(k) = 3.14 * Fuel(k) - 2.015\ M_{SAF}$$

And in 2100 to:

$$CO2(k) = 3.14 * Fuel(k) - 2.617\ M_{SAF}$$

Method for CO_2-trading

Since the top-level scenario assumptions for the CORSIA scenario is a constant CO_2 emission after 2020, the remaining rest, which is not dealt with by SAF has to be traded (*Trade, %*):

$$Trade(k) = \frac{Fuel(k) * \ EICO2(k) - \ CO2(2020) - \ SAF(k) * EI_{SAF}(k).}{CO2(k)}$$

Method for soot particle number reduction

Between 1940 and 2020 no reduction in the emission of soot particle number densities is assumed. From 2021 to 2100, we assume that SAF are used worldwide and have the same blend (equal to *SAF*) worldwide, which reduces the particle number densities in the emissions. We further assume that a 50% blend reduces the particle number densities by 50% and interpolate linearly for other blends.

8) Flightpath 2050 (FP2050 and FP2050-cont)

Interpretation of top-level goals for CO_2 and NO_x emissions

The Flightpath 2050 scenario is largely based on the BAU scenario, with the exception that the CO_2 emissions (and hence fuel use as a consequence) and the NO_x emissions are largely reduced in line with the Flightpath 2050 goals. These goals state for a new aircraft in 2050:

"CO_2 emissions per passenger kilometre have been reduced by 75%, NO_x emissions by 90% and perceived noise by 65%, all relative to the year 2000."[19]

The technologies to achieve these goals are currently developed and we assume that they are introduced into the market in 2050 in the scenario FP2050 and linearly from 2020 onwards in FP2050-cont. We take into account the mean age of the aircraft based on the WeCare scenario. Since the WeCare data are available for 2012 and the mean age of that year is roughly 11 years, we take the year 2012 as representative for the year 2001 technology reference. Since the age of the aircraft decreases to 9 years in the year 2050 in the WeCare scenario (see the respective section), we take the year 2059 as a reference for the year 2050 technology. Hence the two top-level targets can be written as

$$\frac{CO2(2059) / RPK(2059)}{CO2(2012) / RPK(2012)} = 1 - 75\% = 25\% \text{ and}$$

$$\frac{NOx(2059) / RPK(2059)}{NOx(2012) / RPK(2012)} = 1 - 90\% \; = 10\%.$$

Method for Fuel use and CO_2-Emissions

The fuel efficiency improvements (in relative annual changes) for the FP2050 scenarios follow the BAU scenario until 2050 and after 2059. In the phase when the new technologies are introduced and replace older technologies (2050 to 2059) the annual fuel efficiency improvements are 9.68% per year, which results in an achievement of the CO_2 emission reduction goals of 75%. For FP2050-cont, new technologies are introduced much earlier, beginning in 2020 and the annual improvement rate is hence lower, 2.51%/year.

Method for NO_x-Emissions

The NO_x emission index follows the BAU scenario for the years 1940 to 2050. In 2050 new technologies are introduced to achieve the top-level target. A linear decrease of the EI_{NOx} from 14.3 g(NO_2)/kg(fuel) in 2050 to 5.7 g(NO_2)/kg(fuel) is considered, which meets the requirements. After 2059 an unchanging emission index is assumed, following the BAU scenario, though at a lower absolute level. For FP2015-cont, this transition starts in 2020.

9) COVID-19 Scenarios

To estimate uncertainties from the impact of the current pandemic situation, we developed three possible pathways for the past-pandemic evolution. Here three major storylines are used, which are based on the BAU scenario with additional COVID-19 implications (Supplementary Table 6).

Supplementary Table 6: Overview on the COVID-19 scenarios

Short Name	Long Name	Description
**C19-03**	COVID-19 with fast recovery	In the year 2020, the COVID-19 pandemic situation leads to a strong decline in aviation transport, which recovers rapidly within 3 years towards the BAU scenario.
**C19-15**	COVID-19 with slow recovery	In the year 2020, the COVID-19 pandemic situation leads to a strong decline in aviation transport, which recovers slowly within 15 years.
**C19-15s**	COVID-19 with slow recovery and a change in passenger habits	As C19-15, but with a change in habits, e.g. use of Web-Conferences, leading to a sustained decrease in air transportation

ICAO[6] has analyzed the short-term impacts of the current COVID-19 pandemic situation. They came up with 2 different short-term recovery scenarios. A fast recovery with an annual mean decrease of available seats of 45% compared to a baseline (Scenario "V", with "V" indicating a short drop) and a more pessimistic recovery with an annual mean decrease of available seats of 63% compared to a baseline (Scenario "U", with "U" indicating a longer drop). Here we take those numbers for 2020 and allow 3 and 15 years for recovery. The period of the lockdown initiated many more work meetings via the internet. Currently, it is unclear whether this may initiate a sustained change in working habits. Therefore, we also introduce a scenario where face-to-face meetings are replaced by web-conferences and we allow a 20% decrease in RPK after the 15 years of recovery (C19-15s).

[6] Effects of Novel Coronavirus (COVID-19) on Civil Aviation: Economic Impact Analysis, ICAO, 8th May 2020, Air Transportation bureau, Montréal Canada.

Part 2: Development of ECATS technology scenarios

Supplementary material to

Evaluating the climate impact of aviation emission scenarios towards the Paris Agreement including COVID-19 effects

Volker Grewe[1,2,3], Arvind Gangoli Rao[2,3], Tomas Grönstedt[4,3], Carlos Xisto[4,3], Florian Linke[5,3], Joris Melkert[2,3], Jan Middel[6,3], Barbara Ohlenforst[6,3], Simon Blakey[7,8,3], Simon Christie[9,3], Sigrun Matthes[1,3], Katrin Dahlmann[1,3]

[1]Institut für Physik der Atmosphäre, DLR-Oberpfaffenhofen, Weßling, Germany
[2]Delft University of Technology, Faculty of Aerospace Engineering, Delft, Netherlands
[3]ECATS International Association, Brussels, Belgium
[4]Chalmers University of Technology, Mechanics and Maritime Sciences, Gothenburg, Sweden
[5]Lufttransportsysteme, DLR-Hamburg, Germany
[6]Royal Netherlands Aerospace Centre (NLR), Amsterdam, Netherlands
[7]University of Birmingham, Mechanical Engineering, Birmingham, United Kingdom
[8]University of Sheffield, Mechanical Engineering, Low Carbon Combustion Centre, United Kingdom
[9]Manchester Metropolitan University, Department of Natural Sciences, Manchester, United Kingdom

10) Abstract

To estimate the future climate impact of aviation and to relate it to the Paris Agreement targets, a description of future pathways for aviation is necessary. Whereas the general background scenario is taken from Randers[6], in this supplementary material, we explain in detail how we develop technology scenarios in a bottom-up approach. In this way we can verify, from a technical feasibility point of view, scenarios that are developed top-down. The scenarios are created based on expert knowledge on the potential availability and performance of future aircraft technologies. We split the global air traffic market into two segments, single-aisle and twin-aisle. For the single-aisle market, we provide one future pathway based on expected technology improvement factors for the next aircraft generations. For the twin-aisle market, we consider three possible pathways, based on feasibility studies from literature, which differ in aircraft configuration and therefore substantially vary in the underlying technology improvement factors. We describe the scenario assumptions concerning the markets and reference aircraft, followed by a detailed description of how we derive the technology scenarios. Finally, the methodology describes how we use technological assumptions to create global emission scenarios as a basis for estimating the climate impact.

11) Underlying assumptions

When looking at the market of passenger air transport, the market can be segmented into around 8 categories, ranging from small general aviation aircraft and personal air vehicles to long-range four-engine twin-aisle aircraft. However, from a CO_2 emission and climate impact point of view, a coarse division can be made into single-aisle aircraft and twin-aisle aircraft. This is because regional flights (including general aviation, regional turboprops) and business aircraft have a negligible contribution to climate change[20] and are therefore have been ignored for the sake of brevity in this study. From a climate impact point of view, the short to medium range, medium-range and long-range aircraft, cumulatively are responsible for around 95% of the available seat-kilometres[21] and are therefore responsible for the majority of emissions. These segments are currently served with the help of single-aisle and twin-aisle aircraft. Single-aisle aircraft serving short and short-to-medium distance routes are responsible for 47% of the worldwide aviation fuel consumption. Single- and twin-aisle aircraft serving the medium and long-range routes are responsible for another 47% of the fuel consumption. We, therefore, assume that it is sufficient to split global aircraft movements into single-aisle and twin-aisle aircraft market segments and consider them separately.

Assessing the development potential of aviation, with a particular focus on improving energy efficiency, revealed that many of the scenarios being evaluated are defined through goal setting (top down) rather than through estimates based on a bottom-up technology assessment approach. Examples of such ambition driven scenarios are the well-known ACARE Flightpath 2050 and the NASA N+3 goals.

However, several authors have contributed to building at least a partial understanding of how technology can underpin these technological goals. The potential of technology development to address long term U.S. emission goals has been studied by Hileman et al.[20] including the possibilities to domestically produce and use biofuels. Hileman et al.[20] developed a predictive technology scenario on an assumed transition to U.S. single-aisle aircraft usage only, based on an ultra-advanced variant of the Boeing 737-800 aircraft. A double-bubble lifting fuselage configuration[22] using a reduced cruise speed and an aggressive inclusion of technology suggested a 42% reduction in fuel burn. Further

advancement in propulsion and radical airframe technology produced a 69% reduction to the 2010 baseline. Another notable effort, focused on the analysis of CO_2 emission mitigation costs[23], also included an extensive list of technologies suitable for aircraft retrofitting. This suite of technologies was used to develop an intermediate 2016 aircraft producing a 15% fuel burn benefit over the year 2012 reference. For the longer term, the year 2035, the same study predicted an additional 30% in reduced fuel burn over the intermediate aircraft and a total of 40% over the year 2012 reference. Albeit an extensive list of technology options outlined for the 2016 aircraft, an additional 30% improvement predicted for the 2035 aircraft was attributed more broadly to an open rotor propulsion system, a lower flight speed and an all composite structure. However, it should be noted that the work did not discuss non-CO_2 effects from aircraft emissions.

A comparative study of a long-range and a medium-range 2050 aircraft[24] indicated that large improvements in the energy efficiency of long-range aircraft are more difficult to achieve than for medium-range aircraft. This was partly due to a more relaxed cruise Mach number for the medium-range aircraft (Mach 0.72) and that an open rotor architecture could be used, but also due to that the reference aircraft propulsion system for the long-range configuration is already very efficient and hence more difficult to improve upon (Trent772B engine versus the CFM56-7 engine). The comparative study indicated a 45% reduction in energy need for the long-range configuration and a 59% fuel burn reduction for the medium-range aircraft compared to the year 2000 references. By inclusion of ultra-advanced engine cores having constant-volume-combustion, this improvement could be pushed to 54% for the long-range and 68% for the medium-range aircraft. However, the TRL level of this technology is quite low.

The authors argue that there is a need for a thorough technology-based bottom-up study assessing the impact of key technologies, concerning both design range and entry into service. In particular, it has been the focus to assess non-CO_2 emissions on a consistent set of aircraft with matching entry into service years.

12) Reference aircraft

For each segment, we use one best in class representative aircraft type to model the entire market segment. For the single-aisle market, the Airbus A320neo is selected, while for the twin-aisle market the Airbus A350 is selected as a reference aircraft type for the current generation. Entry into service year of the current generation is assumed to be around 2015. As in 2015, most of the aircraft in the fleet were the previous generation of aircraft. We also model this precursor generation using the aircraft types that are predominant in their respective market segment, i.e. the Airbus A320 for single-aisle and the Boeing 777 for the twin-aisle market.

13) Technologies for future generation aircraft programmes

We are considering two future aircraft generations per market segment. The next-generation single-aisle and twin-aisle aircraft are assumed to be entering service in 2035, these will be followed by another next-generation, which is assumed to be introduced in 2050 (for both market segments). We model the improvements per generation in terms of fuel consumption and NO_x emissions reduction potential that were derived as follows:

Fuel consumption reduction potential

When looking at potential technologies that could reduce future fuel consumption and/or could reduce the climate impact of aviation, we can make a split into eight main categories as shown in Supplementary Figure 7.

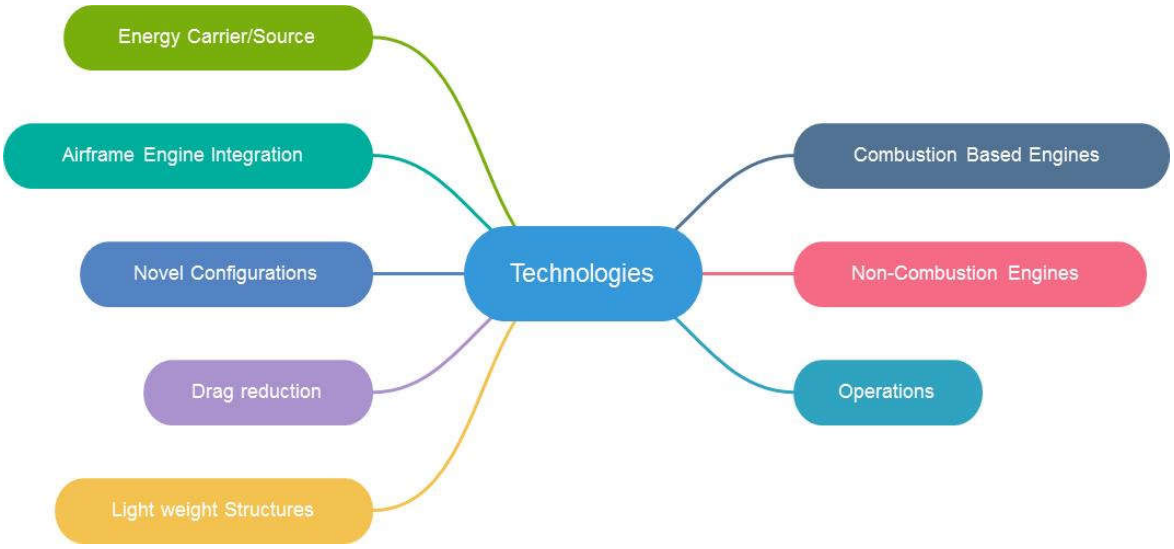

Supplementary Figure 7: Aircraft technologies for future fuel consumption reduction and/or reduction of aviation climate impact

These eight categories can be further refined and assessed for their potential impact. A couple of examples of technologies that could contribute is mentioned below. However, it should be noted that the actual set of technologies is much larger than the ones mentioned below. The detailed breakdown of airframe technologies is shown in Supplementary Figure 8 and that for combustion-based engines is shown in Supplementary Figure 9.

Energy carriers/sources[7]

- Biofuels
- Synthetic fuels
- Batteries
- Liquefied Natural Gas
- Hydrogen

Airframe integration

- Boundary layer ingestion
- Distributed propulsion system

Novel configurations

- Blended wing body aircraft
- Flying-V
- Strut-braced wings
- Double Fuselage

Drag reduction

- Laminar flow wings

[7]Energy sources are added for completeness, but are not further taken into account in the analyses.

- Flow control
- Adaptive wings

Lightweight structures

- Advanced composites
- Smart structures
- Additive manufacturing

Combustion based engines

- Advanced cores
- Variable pitch fan
- Advanced geared turbofan engines

Non-combustion based engines

- Electric motors
- Fuel cells
- High-density power electronics

Operations

- 4-D navigation
- Formation flying
- Flying lower and slower

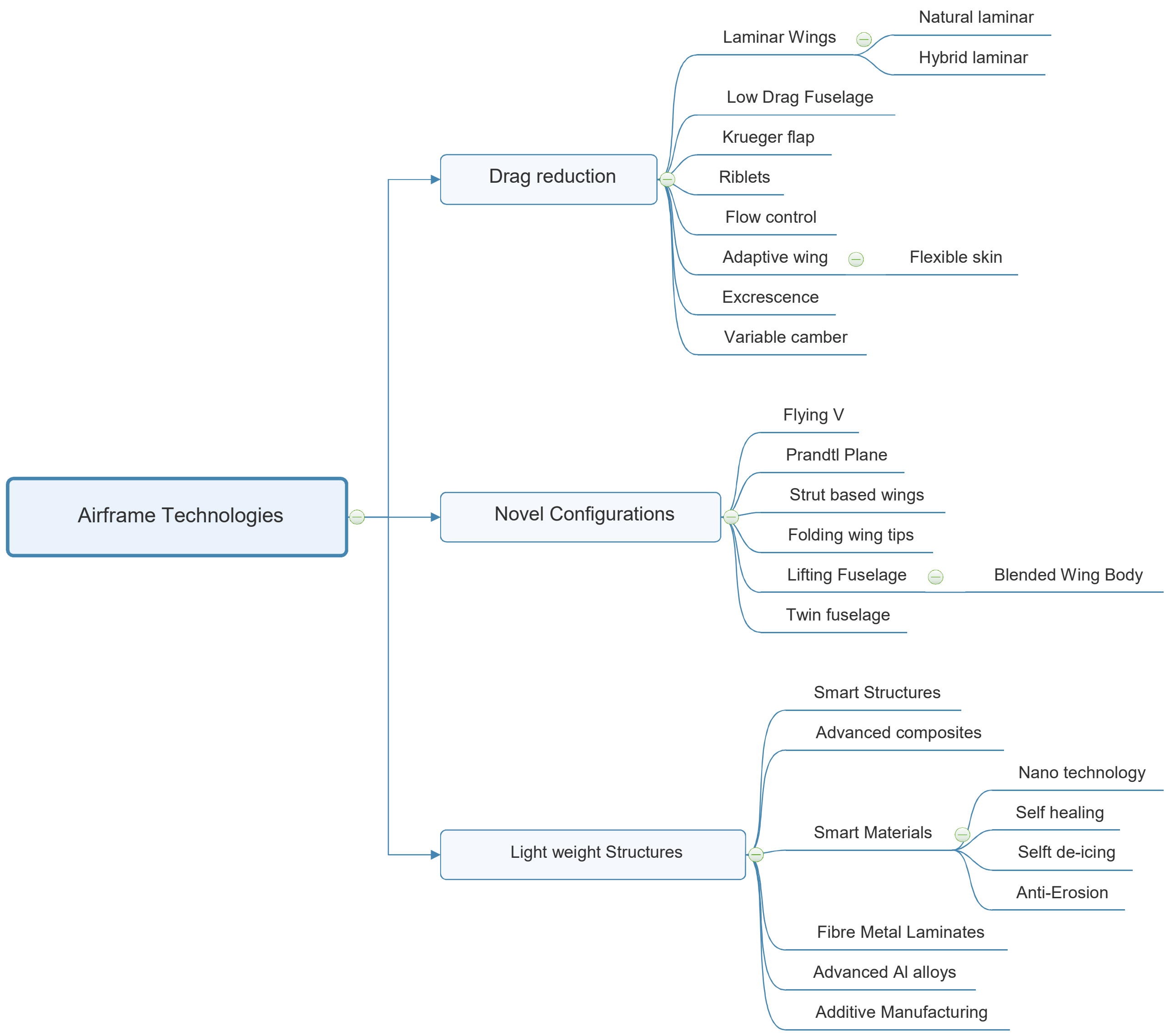

Supplementary Figure 8: Airframe technologies considered for technological assessment in the current study.

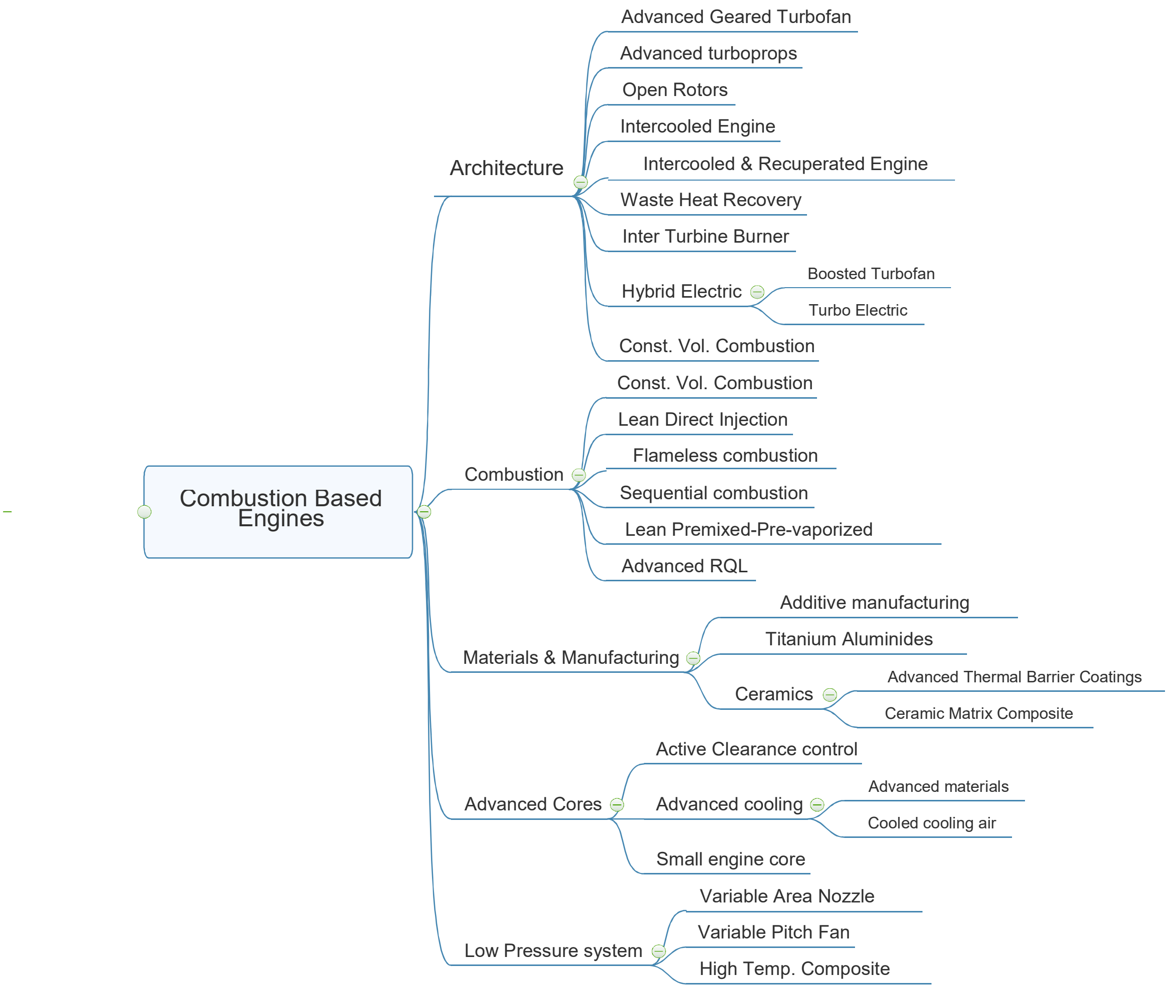

Supplementary Figure 9: Combustion based aero engine technologies considered for technological assessment in the current study.

Analysis Methods: For evaluating the decrease in engine specific fuel consumption, engine models such as GSP (Gasturbine Simulation Model) from NLR and TU Delft[25] and GESTPAN from Chalmers University of Technology[26] have been used. From evaluation of the literature, it is clear that hybrid-electric architecture is not suitable for Single-Aisle or Twin-Aisle aircraft due to the poor energy densities of batteries, lower power densities of electrical components and their associated thermal management issues[27]. The potential for engine improvements from cycle changes has been investigated in the Ultimate Project[24] and in the AHEAD project[28,29].

With respect to evaluating the effect of individual technologies, several factors have to be taken into account as many technologies have a non-linear effect on the overall fuel consumption and emissions of the aircraft. For example: the increase in the engine pressure ratio and by-pass ratio increases engine nacelle drag, engine weight and might have an impact in the aircraft landing gear. The sensitivity of the single-aisle reference aircraft parameters to change in engine specific fuel consumption (SFC) and engine weight and size for a single aisle aircraft are shown in the Supplementary Figures 10 and 11, respectively. They show that while some parameters (like engine SFC) have a significant impact on the aircraft fuel burn, engine weight and size have a limited influence. This analysis has been carried out by using an in-house aircraft design tool called as the Initiator[30].

Operative Empty Mass = 42.913 tons

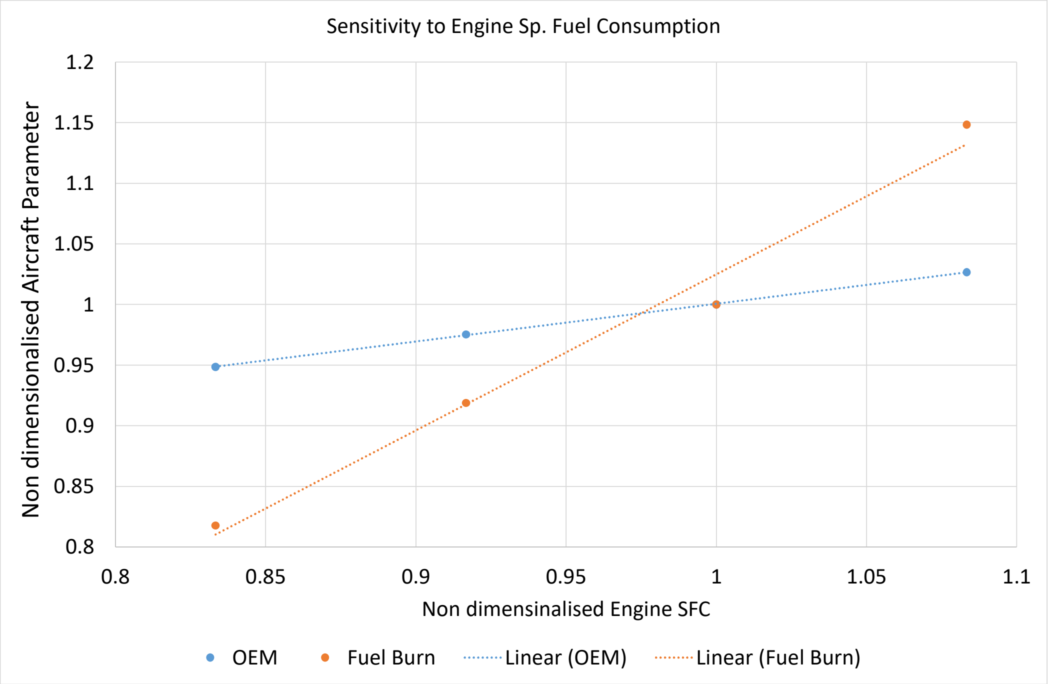

Supplementary Figure 10: Sensitivity of aircraft fuel burn and aircraft operating empty weight to engine specific fuel consumption (obtained from the sensitivity studies carried out using in-house tools)

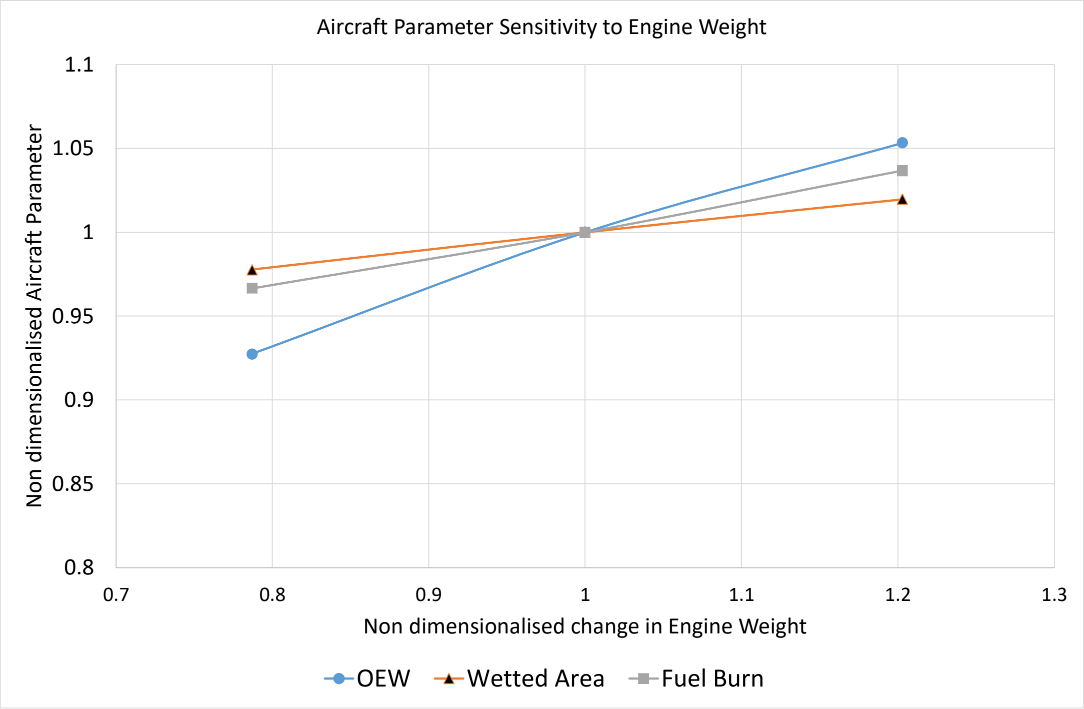

Supplementary Figure 11: Sensitivity of aircraft design parameters to a change in engine weight and nacelle wetted area (obtained from the sensitivity studies carried out using in-house tools).

The sensitivity studies were used to evaluate snow ball factors to assess the likelihood of individual technologies on the aircraft using the aircraft performance simulator Piano-X. A similar (but much simpler) approach was used in the study carried out by Air Transportation Analytics Ltd and Ellondee Ltd for the Committee on Climate Change and the Department for Transport of the UK government in 2018[31].

The set of technologies considered in evaluating aircraft fuel reduction was based on the likelihood of these technologies being used on that aircraft and their respective TRL levels. The suite of technological improvements considered for the next generation of single-aisle aircraft (SA-2035) is graphically shown in Supplementary Figure 12. It should be noted that the aggregated efficiencies for each of the sub categories are not summative in nature due to law of diminishing return and their non-linear influence on the aircraft level fuel burn. A similar graphical schematic of technological suite that could be used for a conventional tube and wing based single-aisle aircraft (SA-2050) and a twin-aisle aircraft in year 2050 (TA-2050) are shown in Supplementary Figures 13 and 14, respectively.

There is a possibility that in the year 2050, a non-conventional aircraft configuration could be used for the long-range mission. Such configurations have been studied by a number of researchers. For the sake of brevity, two studies have been shown here, the first aircraft represents a futuristic radical configuration based on a combustion based gas turbine propulsion system (represented by the blended wing body designed within the AHEAD project[32,33] and Flying V[34,35] carried out at the Delft University of Technology) and the second aircraft represents a future radical aircraft based on turbo-electric propulsion system, such as the NASA N3-X[36,37]. It should be noted that the probability of such aircraft making into the market is low due to the associated high development costs and risks. Nevertheless, these concepts show a glimpse of what could be possible.

Supplementary Table 7 provides an overview of the possible increase in efficiency that can be obtained by the various aircraft types discussed above.

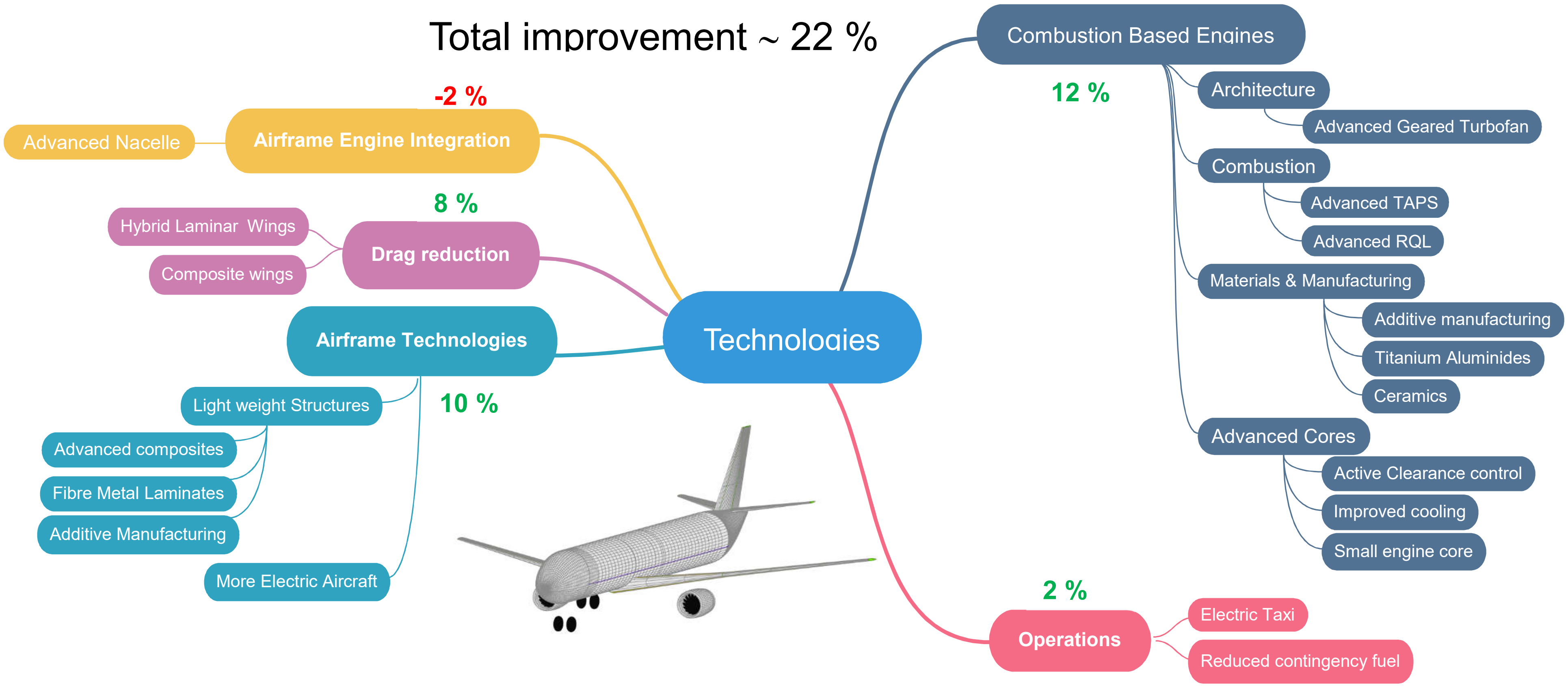

Supplementary Figure 12: Suite of projected technologies considered and their aggregated contribution in increasing efficiency of a Single-Aisle aircraft for the year 2035.

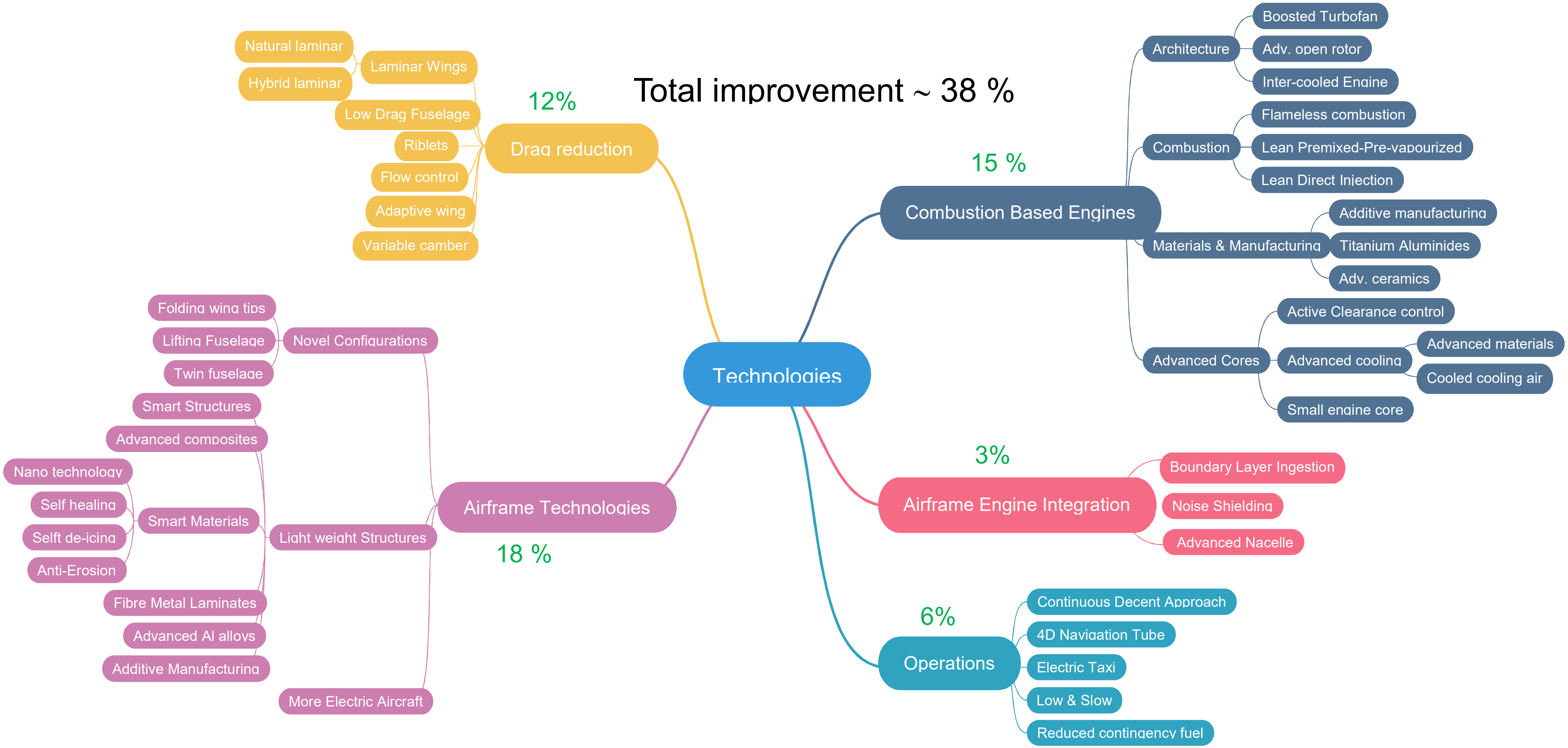

Supplementary Figure 13: Suite of projected technologies considered and their aggregated contribution in increasing efficiency of a Single-Aisle aircraft for the year 2050.

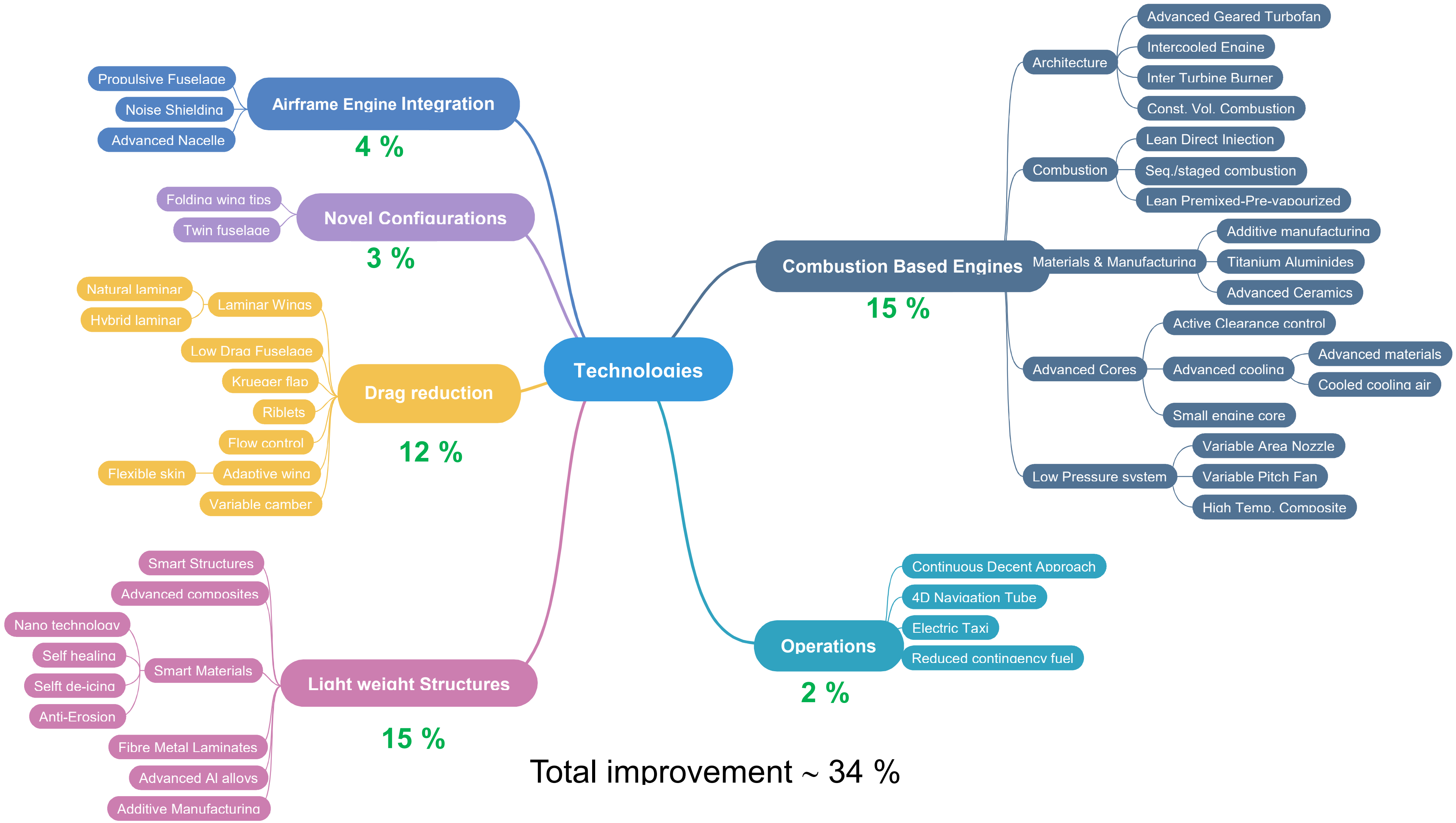

Supplementary Figure 14: Suite of projected technologies considered and their aggregated contribution in increasing efficiency of a twin-aisle aircraft for the year 2050.

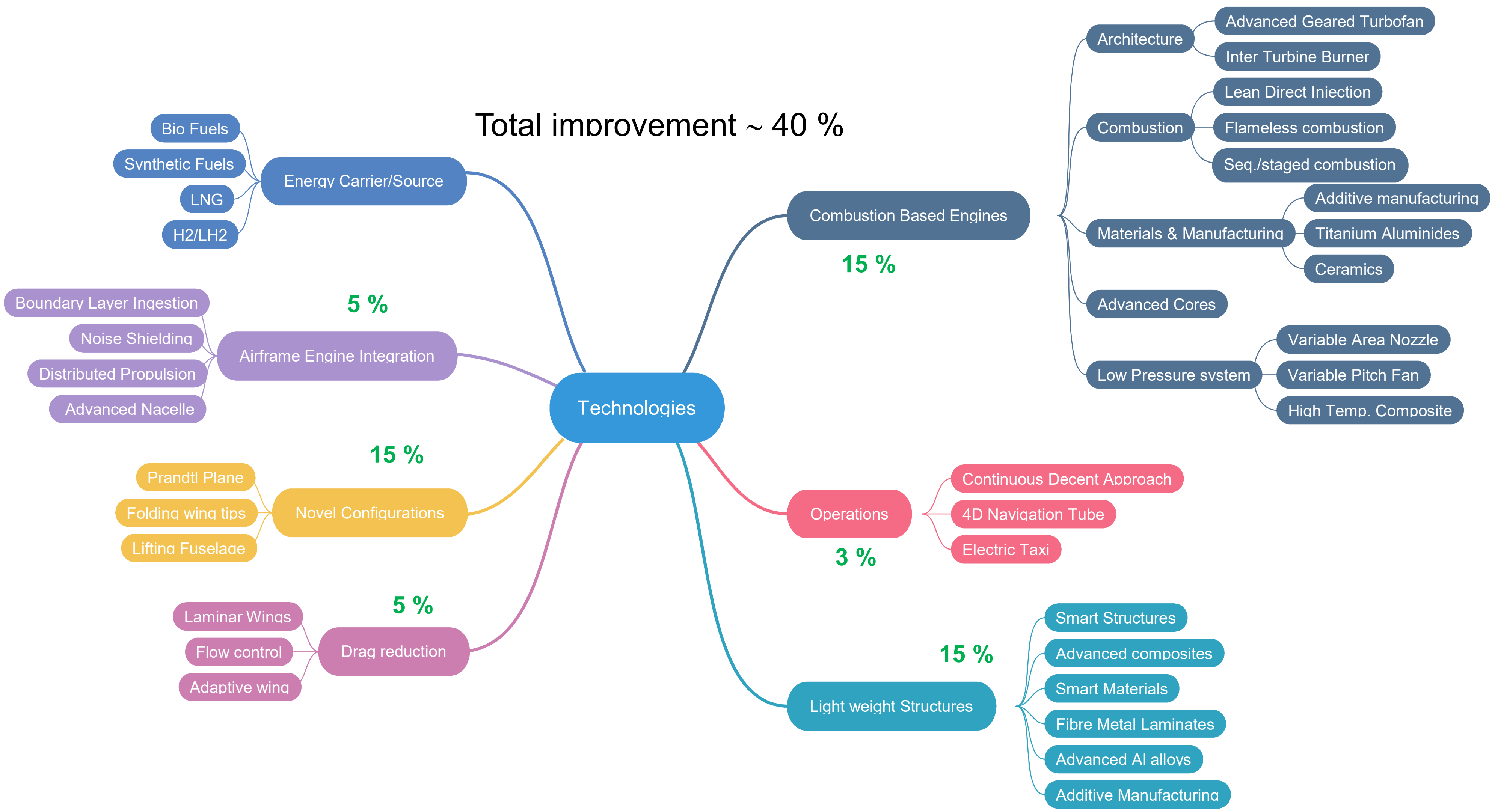

Supplementary Figure 15: Suite of projected technologies considered and their aggregated contribution in increasing efficiency of radical aircraft with turbofan engine and Boundary Layer Interaction (BLI) for the year 2050.

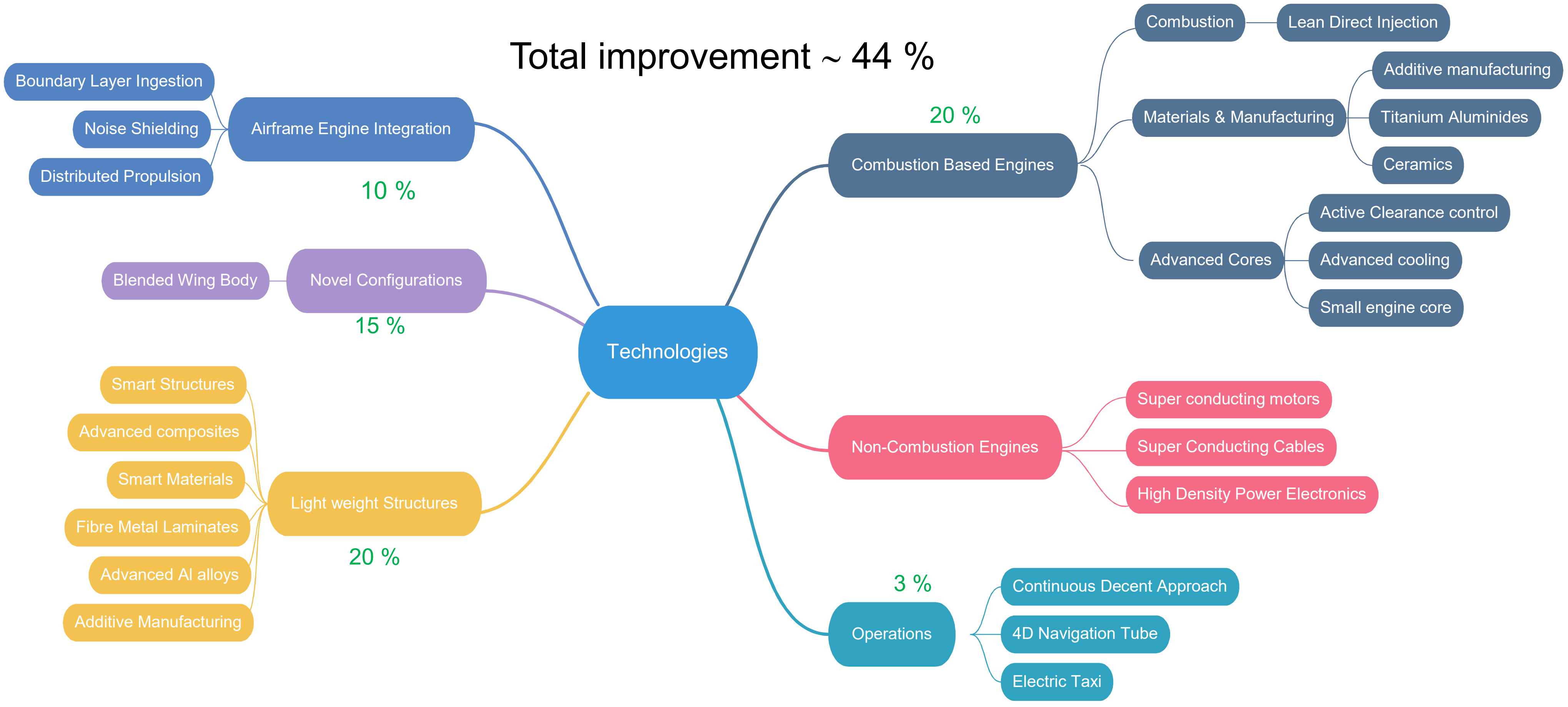

Supplementary Figure 16: Suite of projected technologies considered and their aggregated contribution for a radical aircraft with turbo electric propulsion and BLI for the year 2050.

Supplementary Table 7: Estimate of potential future fuel consumption reduction with respect to A320neo (single-aisle) and A350 (twin-aisle).

	Next Generation 2035		Future Generation 2050			
Technology	Single-aisle	Twin-aisle	Single-aisle	Twin-aisle (long-range)		
				Conven-tional Tube and Wing Config.	Novel aircraft	
					Turbofan Flying V and MF-BWB	Turboelec. propulsion NASA N3-X
Airframe engine integration	+2%	+1%	-3%	-4%	-5%	-10%
Novel configurations	--	--	--	--	-15%	-15%
Drag reduction	-8%	-6%	-12%	-10%	-5%	--
Lightweight structures	-12%	-10%	-18%	-15%	-15%	-20%
Combustion based engines	-10%	-8%	-16%	-14%	-15%	-20%
Operations	-2%	-1%	-4%	-3%	-3%	-3%
Total estimated improvement	-22%	-18%	-38%	-34%	-40%	-44%

In Supplementary Table 7, we have not included changes in energy carriers/sources and non-combustion engines. The total estimated fuel burn improvement is not the direct sum of all individual contributions. We have considered the integration effects and the "snowball effect".

Comparing with the work by Schäfer et al.[23] the improvement rates are quite similar when matching the 2035 single-aisle aircraft with the "evolutionary" year 2035 configuration presented by Schäfer. The reference used in this paper is more recent and is comparable to Schäfer's "intermediate" aircraft. The work predicts an 18% fuel burn reduction of the evolutionary aircraft over the intermediate aircraft, which is similar to that obtained in our analysis. The difference is within the uncertainty range of assumptions made regarding design cruise speed, which is not stated in the work by Schäfer[23]. A direct comparison with Hileman's work[20] is not possible since its first evolution step assumes transition to a double-bubble configuration, whereas herein a more conservative tube-and-wing architecture is assumed for short to medium range single-aisle aircraft. A more direct comparison with the ULTIMATE project year 2050 tube and wing long range configuration[24] is possible. For this configuration a fuel burn reduction of 45% was predicted over a year 2000 reference. Correcting for the more recent reference stated in this paper this gives that the ULTIMATE long-range configuration would provide a 35% fuel burn reduction, a result that matches well with the bottom-up prediction presented here.

For climate assessment, the aircraft fuel burn is a good indicator of the associated CO_2 and H_2O emission however other engine emissions, such as NO_x emission, is a function of several engine and combustor design and operating parameters. The subsequent section deals with the NOx emission potential for the future engines, which are then used in the bottom-up scenario to estimate the climate impact of aviation.

NO_x emissions reduction potential

State-of-the-art

The in-service state-of-the-art combustion technology in 2020 comprises of advanced Rich-Quench-Lean (RQL) and lean combustion. The working principle of RQL is to avoid stoichiometric combustion by burning with excess fuel in the primary zone, followed by a rapid mix with dilution air in the quench section and finally by burning the remaining fuel with excess air in the lean region[38]. Combustion with excess fuel reduces NO_x primarily due to the competition between fuel and nitrogen for oxygen. In RQL combustion most of the air is employed for dilution (approx. 45%) followed by cooling (30%) leaving only 25% of air available for combustion in the primary region[39]. In contrast to RQL, lean technology utilizes almost all the available air for combustion (approx. 70%) leaving the remaining 30% for cooling. Combustion with excess air results in moderate flame temperatures and low NO_x emissions.

For RQL, the state-of-the-art combustion technology is provided by Pratt & Whitney with their TALON X combustor and by Rolls-Royce with their Phase 5 RQL tiled concept. The TALON X powers the geared turbofan for the short-medium range aircraft, whereas Phase 5 is offered in Rolls-Royce' entire family of large civil turbofan engines.

Lean combustion is represented by the General Electric (GE) Twin Annular Premixing Swirler (TAPS) combustor. The TAPS combustor is comprised of a central pilot injector with a concentrically mounted main. During ignition up to idle only the pilot is operated, whereas at moderate to high power the main is turned on as well. This type of staging technology can be optimized to reduce emissions in each segment (e.g. the land and take-off cycle below 3000 ft) or in the entire mission[40]. TAPS was developed for the GE GEnx large turbofan engine powering the twin-aisle Boeing 787 and also integrated into the

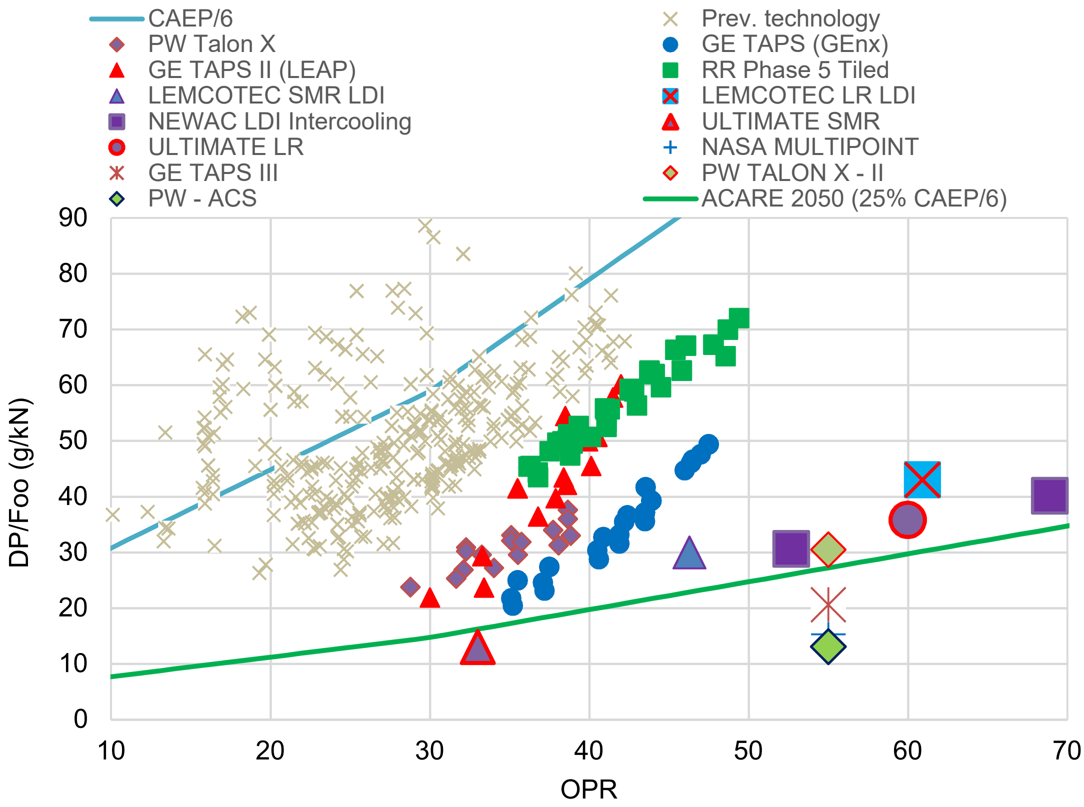

Supplementary Figure 17: ICAO LTO NO_x emissions of in service and research combustion technology.

CFM Leap engine powering the Airbus A320neo family. The year 2020 state-of-the-art combustors are found in Supplementary Figure 17 below together with a collection of future combustor concepts.

Supplementary Table 8: ICAO LTO cycle NO_x emission data publicly available from previous and ongoing research programmes in Europe and US.

	OPR	Dp/Foo [g/kN]	Margin to CAEP/6	Improvement margin to CAEP/6 relative to ref	Ref.	Application	TRL
NEWAC - IC - LR	69	40.2	71%	26%	TAPS	2050 (Long-range)	TRL2
NEWAC - IC - SR	53	30.6	71%	26%	TAPS	2050 (Single-aisle)	TRL2
LEMCOTEC SMR LDI	46	30.0	67%	21%	TALON X	2035 (Single-aisle)	TRL5
LEMCOTEC LR LDI	61	43.0	64%	15%	TAPS	2035 (Long-range)	TRL6
GE TAPS III	55	21.0	81%	45%	TAPS	2035-2050 (Long-range)	TRL4
PW TALON X - II	55	30.5	72%	29%	TALON X	2035 (Single-aisle)	TRL4-5
PW - ACS	55	13.1	88%	58%	TALON X	2050 (Single-aisle)	TRL4
ULTIMATE SMR	33	13.0	80%	44%	TALON X	2050 (Single-aisle)	TRL2
ULTIMATE LR	60	35.8	70%	25%	TAPS	2050 (Long-range)	TRL2
NASA MULTIPOINT	55	15.3	86%	53%	TAPS	2050 (Long-range)	TRL3

Future low NOx technology

Publicly available data from major projects in Europe and the US is compiled in Supplementary Table 8. In Europe, Rolls-Royce performed TRL 6 demonstrations of Lean Direct Injection (LDI) technology within Clean Sky, LEMCOTEC, and ALECSYS development programmes. In LEMCOTEC, a 64% reduction of NO_x against CAEP/6 was demonstrated[41] (see also Supplementary Figure 17). This technology is expected to go into service in the next generation of Rolls-Royce engines, namely the 3-shaft Advance and geared Ultrafan turbofans. In the FP6-NEWAC programme, the effect of intercooling in NO_x emissions was investigated[42]. The reduction in flame temperature because of lower inlet temperatures due to intercooling could lead to a reduction in NO_x. This effect can be observed by comparing the LEMCOTEC LR LDI (non-intercooled) and NEWAC LDI Intercooled combustors in Supplementary Figure 17.

In the US, Pratt & Whitney is continuing its development of the RQL TALON X combustor within Phase-I and Phase-II of the Environmental Responsible Aviation (ERA) programme. Improvements were introduced into the swirler and front end of the combustor to reduce smoke and NO_x emissions. TRL 4 results from Phase-I revealed that the improved Talon X (Talon X – II in Supplementary Figure 17 and Supplementary Table 8) results in a margin of 72% to CAEP/6, which is an improvement of 29% relative to its predecessor[43]. In the same program, Pratt & Whitney is also investigating the Axially Controlled Stoichiometric (ACS) combustor. The working principle is similar to the TAPS concept, but now the pilot

and main are axially staged. Again, results from ERA Phase-1 at TRL 4 revealed that the ACS has the potential to achieve a margin to CAEP/6 of 88%, improving the margin of the in-service TALON X by 58%.

For the next generation of the TAPS combustor, GE is introducing improvements to increase the fraction of air used for combustion going beyond the 70% used by TAPS I & II, as well as to improve fuel-air mixing with new swirler technology. Ceramic Matrix Composites (CMCs) with improved cooling technology is being investigated in the NASA ERA program to reduce the need for cooling while meeting durability standards. TRL 4 results from Phase-I reveal that the improved TAPS (TAPS III in Supplementary Figure 17 and Supplementary Table 8) gives an 81% margin relative to CAEP/6, which results in an improvement of 45% relative to its predecessor[43].

NASA is also developing its multipoint injection lean combustion concept, where the large single-injector is split into a grid of fuel injectors and air swirlers with a single larger pilot injector in its center[44]. The concept is aiming to improve mixing at a lower pressure loss, relative to a single-injector design. At the same time, stability is also improved with larger quantities of air due to the rapid local mixing. Several variants of this concept were investigated at TRL 3 with margins relative to CAEP/6 up to 86%, improving the margin of the in-service Lean TAPS combustor by 53%[44].

Mission NO_x emission estimates

The NO_x data presented so far are concerned with LTO emissions (below 3000 ft). Estimating mission NO_x is subject to greater uncertainty due to the lack of regulation- and certification data. The analysis relies on emission correlations for state-of-the-art and future combustor types[44,45], supplemented by aircraft performance analysis computations. Within the EU H2020 project ULTIMATE[24], airframe and engine technology with the year 2050 as a horizon was set up and optimized. Mission NO_x was estimated by applying correlations for LDI combustion technology[45] supplemented by the year 2050 aircraft and propulsion performance data[24,46]. The benefits expected from the low NO_x combustor technology were cancelled almost exactly by the more aggressive cycles adopted for reduced CO_2 emissions. Hence, the reported reductions in NO_x emissions are equal to the reduction in fuel burn. Second generation lean combustion, for the year 2035 turbofan, is expected to reduce mission NO_x beyond fuel burn improvements by 40%[47] due to the introduction of ceramic matrix composite liners and improved fuel-air mixing systems. However, the uncertainty is still high since those systems were demonstrated during NASA Phase - I at TRL 4 in 2009, and results at TRL5 from Phase-II in 2015 are not yet available in the public domain. Furthermore, cycle data for these engines is expected to become more aggressive as well, which will reduce the potential benefit. A conservative estimate for the mission NO_x reduction is then to assume that it equals the fuel burn reduction. Supplementary Table 9 lists the estimated mission NO_x reductions for the single- and twin-aisle configurations targeting the year 2035 and year 2050 entry into service.

Supplementary Table 9: Estimation of NO_x reductions for each of the proposed aircraft variants.

	Next generation single-aisle (2035)	Next generation twin-aisle (2035)	Single-aisle (2050)	Twin-aisle (2050)	Long-range (AHEAD/Flying-V) (2050)	Long-range (NASA N3-X)(2050)
Mission fuel burn improvement	Appr. -22%	Appr. -18%	Appr.-38%	Appr.-34%	Appr. -40%	Appr. -44%
LTO NO_x (improved margin to CAEP6)	-22% to -26%	-18% to -22%	-25% to -50%	-25% to -50%	-25% to -50%	-25% to -50%
Mission NO_x Imrovement	-22% to -26%	-18% to -22%	-38%	25% to -34%	-30% to -40%	-33% to -44%

14) Aviation growth

The increase of flight movements, as well as the geographic distribution of the flight routes, is derived from the WeCare flight movement inventory[1]. We use data available by seat category for the years 2015 to 2050 from categories 3 to 7 covering all aircraft types between 101 and 600 seats. We assign Cat3 completely to the single-aisle segment as well as all flights shorter than 2,500 NM from Cat4 and Cat5. All remaining flights are assumed to be carried out by the twin-aisle aircraft, i.e. all flights longer than 2,500 NM from Cat4 and Cat5 plus all Cat6 and Cat7 flights (see also Supplementary Table 10).

Supplementary Table 10: Classification of seat categories in the WeCare data set.

**WeCare category**		**Reference aircraft type** Flights ≤ 2,500 NM	> 2,500 NM
Cat3	101-151 seats	A320neo	
Cat4	152-201 seats	A320neo	A350-900[8]
Cat5	202-251 seats	A320neo	A350-900
Cat6	251-301 seats	A350-900	
Cat7	302-600 seats	A350-900	

The flight plans for the years 2055, 2060, 2065 and 2070 are created by extrapolating the 2050 WeCare data according to the trends for single-aisle and twin-aisle aircraft fleets derived from the WeCare data of the years 2015 to 2050. A second-order polynomial parametrization is applied. While we observe a slight progressive development in the flight numbers of the twin-aisle segment, the single-aisle segment shows a digressive behaviour well reflecting the tendency towards larger aircraft in the future (see Supplementary Figure 18).

[8] In general, a more appropriate representative of this class might be A321, however, only 0.02% of all Cat4 flights are longer than 2500 NM and we use the same aircraft for Cat5 for simplicity reason.

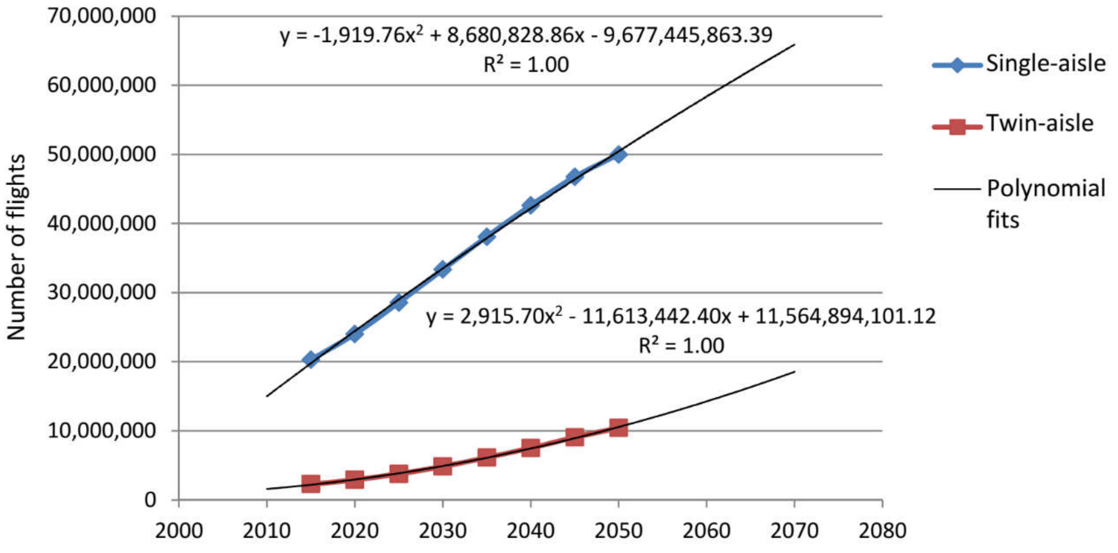

Supplementary Figure 18: Temporal evolution of the number of flights for the single-aisle segment and twin-aisle segment. Data is based on the WeCare project.

Hence, from 2050 onwards, the geographic distribution of routes remains constant and only the flight frequency changes in our model.

15) Fleet diffusion

According to Bass[48], the introduction of new technologies into a market typically follows an S-shaped diffusion curve. We adopt the Bass diffusion model also to describe the temporal development of the market penetration of the next aircraft generations in their respective market segment. Mathematically, the following relationship is used:

$$F(t) = \frac{1 - e^{-(p+q)\cdot t}}{1 + \frac{q}{p} \cdot e^{-(p+q)\cdot t}}$$

The coefficients p=0.03 and q=0.38 are chosen such that the S-curve leads to a market share of approximately 98% within 15 years, starting from the respective year of entry into service (EIS). The resulting development of the market shares is depicted in Supplementary Figure 19. In Supplementary Table 11 the associated values used for the composition of the emission inventories considering co-existence of multiple generations are provided.

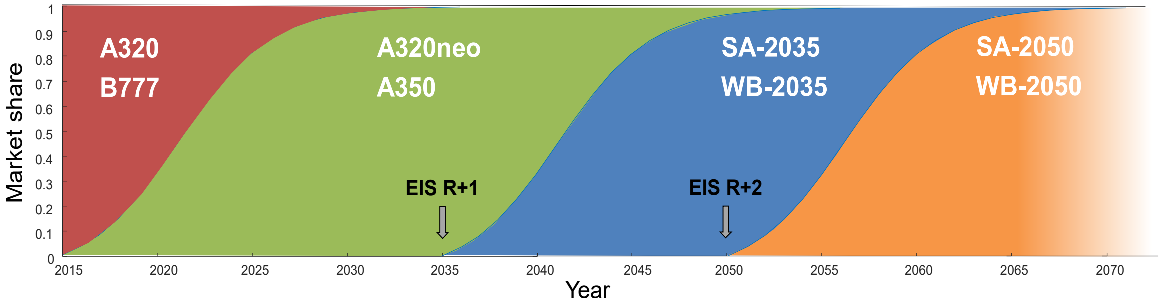

Supplementary Figure 19: Temporal evolution of the market share of aircraft segments.

Supplementary Table 11: Market share for different generations of aircraft between 2015 and 2070.

	2015	2020	2025	2030	2035	2040
**R-1**	100,00%	66,88%	18,72%	2,84%	0,37%	0,05%
**R (2015)**	0,00%	33,12%	81,28%	97,16%	99,63%	66,83%
**R+1 (2035)**	0,00%	0,00%	0,00%	0,00%	0,00%	33,12%
**R+2 (2050)**	0,00%	0,00%	0,00%	0,00%	0,00%	0,00%

	2045	2050	2055	2060	2065	2070
**R-1**	0,01%	0,00%	0,00%	0,00%	0,00%	0,00%
**R (2015)**	18,71%	2,84%	0,37%	0,05%	0,01%	0,00%
**R+1 (2035)**	81,28%	97,16%	66,51%	18,67%	2,83%	0,37%
**R+2 (2050)**	0,00%	0,00%	33,12%	81,28%	97,16%	99,63%

16) Emission distribution from 2015 to 2070

The annual 3D emission inventories are composed by mixing the emission distributions of the different generations according to their market share (weighted sum). For this purpose, we create the underlying emission inventories for each year and each aircraft generation. For the generations, R-1 and R, global emission distributions are calculated using the GRIDLAB methodology[49]. This includes a database of reduced emission profiles that have been prepared by simulating missions with combinations of payload and flight distance covering the complete payload-range envelope of the respective reference aircraft types (see above) with a trajectory simulator. Therein, advanced aircraft performance models from EUROCONTROL's Base of Aircraft Data (BADA V4.2[9])[50] are used in combination with the Total-Energy-Model (see e.g. Nuic et al.[50]) which provides simplified relationships to determine the flight mechanics. Along the trajectories, the gaseous emissions are determined based on the fuel flow of the engine and stored in relation to the trajectory parameters (time, flown distance, altitude). The emissions are computed by multiplying the fuel flow with the Emission Index (EI) of the respective emission species. For CO_2 and H_2O a constant EI of $EICO_2 = 3.14$ and $EIH_2O = 1.23$, respectively, is used. For NO_x emissions the EINOx is calculated as the average of the

[9] https://simulations.eurocontrol.int/solutions/bada-aircraft-performance-model/

two values determined using different state-of-the-art methods, viz. the Boeing Fuel Flow Method 2[12] and the DLR Fuel Flow Method[51]. The reference emission inventories are then generated by processing the entire route network from the growth scenario (see above), for each flight obtain the appropriate reduced emission profile from the database and geographically map it to the flight route. The inventories for the next generations, R+1 and R+2, are created by multiplying the reference inventories R with the improvement factors (CO_2 and H_2O inventories scaled according to fuel improvement, NO_x inventory scaled according to NO_x improvement). Finally, the inventories of the different aircraft generations are superposed according to their respective market share.

17) Supplementary References

1. Grewe, V., Dahlmann, K., Flink, J., Frömming, C., Ghosh, R., Gierens, K., Heller, R., Hendricks, J., Jöckel, P., Kaufmann, S., Kölker, K., Linke, F., Luchkova, T., Lührs, B., van Manen, J., Matthes, S., Minikin, A., Niklaß, M., Plohr, M., Righi, M., Rosanka, S., Schmitt, A., Schumann, U., Terekhov, I., Unterstrasser, S., Vázquez-Navarro, M., Voigt, C., Wicke, K., Yamashita, H., Zahn, A., Ziereis, H., Mitigating the Climate Impact from Aviation: Achievements and Results of the DLR WeCare Project, Aerospace 4(3), 34; doi:10.3390/aerospace4030034, 1-50, 2017.
2. Ghosh, R.; Kölker, K.; Terekhov, T. Future passenger air traffic modelling: A theoretical concept to integrate quality of travel, cost of travel and capacity constraints. In Proceedings of the 19th World Conference of the Air Transport Research Society (ATRS), Singapore, 2–4 July 2015.
3. Ghosh, R.; Wicke, K.; Kölker, K.; Terekhov, I.; Linke, F.; Niklaß, M.; Lührs, B.; Grewe, V. An integrated modelling approach for climate impact assessments in the future air transportation system—Findings from the WeCare project. In Proceedings of the 2nd ECATS Conference, Athens, Greece, 7–9 November 2016.
4. Terekhov, I.; Ghosh, R.; Gollnick, V. A concept of forecasting origin-destination air passenger demand between global city pairs using future socio-economic development scenarios. In Proceedings of the 53rd AIAA Aerospace Sciences Meeting, Kissimmee, FL, USA, 5–9 January 2015.
5. Terekhov, I.; Evans, A.; Gollnick, V. Forecasting a global air passenger demand network using weighted similarity-based algorithms. In Complex Networks VII; Springer: Berlin/Heidelberg, Germany, 2016; pp. 335–347, 2016.
6. Randers, J., 2012: A Global Forecast for the Next Forty Years, Chelsea Green Publishing: White River Junction, VT, USA, ISBN-10: 1603584218, ISBN-13: 978-1603584210, 2012.
7. Hughes, B.; Hillebrand, E. Exploring and Shaping International Futures; Paradigm Publishers: New York, NY, USA, 2006.
8. Kölker, K.; Bießlich, P.; Lütjens, K. From passenger growth to aircraft movements. J. Air Transp. Manag. 2016, 56, 99–106, 2016.
9. Apffelstaedt, A.; Langhans, S.; Gollnick, V. Identifying carbon dioxide reducing aircraft technologies and estimating their impact on global CO2 emissions. In Proceedings of the Deutscher Luft- und Raumfahrt Kongress (DLRK), Aachen, Germany, 8–10 September 2009.
10. Kölker, K.; Ghosh, R.; Lütjens, K. Assessing quality of air travel using the impact of frequency, travel time and transfers on passenger demand. In Proceedings of the 19th World Conference of the Air Transport Research Society (ATRS), Singapore, 2–4 July 2015.
11. Linke, F. Ökologische Analyse Operationeller Lufttransportkonzepte. Ph.D. Thesis, Hamburg University of Technology (TUHH): Hamburg, Germany, 2016.
12. DuBois, D., Paynter, G.C.: Fuel Flow Method2 for Estimating Aircraft Emissions, Society of Automotive Engineers (SAE) 2006-01-1987, 2006.
13. International Civil Aviation Organization (ICAO): ICAO Engine Exhaust Emissions Databank, ICAO Doc 9646-AN/943, First Edition 1995 (plus revisions from

https://www.easa.europa.eu/easa-and-you/environment/icao-aircraft-engine-emissions-databank), Last access: 22 July, 2019
14. Cooper, T.; Smiley, J.; Porter, C.; Precourt, C. Oliver Wyman Global Fleet & MRO Market Forecast Summary 2017-2027, 2017.
15. UK, The Energy Institute and Coordinating Research Council, The Quality of Aviation Fuel Available in the United Kingdom Annual Survey 2014, ISBN: 9780852938379, published 2018.
16. Blakey, S., Wilson, C.W., Farmery, M., Midgley, R., Fuel effects on range versus payload formodern jet aircraft, The Aeronautical Journal 115, 627-634, 2011.
17. PQIS, Defence Energy Support Center, Petroleum Quality Information System Annual Report 2008, published 2008.
18. EU, DIRECTIVE (EU) 2018/2001 OF THE EUROPEAN PARLIAMENT AND OF THE COUNCIL, of 11 December 2018 on the promotion of the use of energy from renewable sources (recast) https://eur-lex.europa.eu/legal-content/EN/TXT/PDF/?uri=CELEX:32018L2001&from=EN , 2018.
19. ACARE, Strategic Research& Innovation Agenda Volume 1, 2012.
20. Hileman, J. I., Rosa Blanco, E. D. l., Bonnefoy, P. A., Carter N. A., The carbon dioxide challenge facing aviation, Progress in Aerospace Sciences, vol 63, pp. 84-95, 2013.
21. Yutko, B.M. and Hansman, R.J., Approaches to representing aircraft fuel efficiency performance for the purpose of a commercial aircraft certification standard, M.Sc., Massachusetts Institute of Technology, 2011.
22. Drela, M. Design Drivers of energy-efficient transport aircraft, SAE International Journal of Aerospace, 2011; 4(2) 602–618, http://dx.doi.org/10.4271/2011-01-2495.
23. Schäfer, A., Evans, A., Reynolds, T. et al. Costs of mitigating CO_2 emissions from passenger aircraft. Nature Climate Change 6, 412–417, DOI:10.1038/nclimate2865, 2016.
24. Grönstedt, T., C. Xisto, V. Sethi, A. Rolt, N. G. Rosa, A. Seitz, D. Missirlis, J. Whurr, N. Tantot, M. Dietz and A. Lundbladh, Conceptual design of ultra-efficient cores for mid-century aircraft turbine engines, in ISABE 2019-24335, Canberra, Australia, 2019.
25. Visser,W., Generic Analysis Methods for Gas Turbine Performance, PhD Thesis, TU Delft, 2015.
26. Grönstedt, T., Development of Methods for Analysis and Optimization of Complex Jet Engine Systems, PhD Thesis, Chalmers University, 2000.
27. van Holsteijn, M.R., Gangoli Rao, A., and Yin, F., Operating characteristics of an electrically assisted turbofan engine, GT2020-15355, Proceedings of Proceedings of ASME Turbo Expo 2020: Turbine Technical Conference and Exposition, Virtual conference, September 2020.
28. Yin, F. and Gangoli Rao, A., Performance Analysis of an Aero Engine with Interstage Turbine Burner, The Aeronautical Journal, Vol. 121 (1245), pp. 1605-1626, 2017.
29. Yin, F., Gangoli Rao, A., Bhat, A. & Chen, M., Performance assessment of a multi-fuel hybrid engine for future aircraft, Aerospace Science and Technology. 77, p. 217-227, 2018.
30. Elmendorp, R.J.M., Vos, R., and La Rocca, G., A conceptual design and analysis method for conventional and unconventional airplanes, ICAS 2014: Proceedings of the 29th Congress of the International Council of Aeronautics. St. Petersburg, Russia, 7-12 September 2014.
31. Air Transportation Analytics Ltd. and Ellondee Ltd., Understanding the potential and costs for reducing UK aviation emissions, Report to the Committee on Climate Change and the Department for Transport. November 2018.
32. Gangoli Rao, A., AHEAD: Paving the way for Next Generation Aircraft and Engine, paper 4B-1, Aero Days, European Commission, 20-22 October, London 2015.
33. Hageman. R., Rudder Incorporated Winglet Design for a Blended Wing Body Aircraft, MSc Thesis, Delft University of Technology, 2016.
34. Faggiano, Francesco; Vos, Roelof; Baan, Max; van Dijk, Reinier, Aerodynamic Design of a Flying V Aircraft, 17th AIAA Aviation Technology, Integration, and Operations Conference: 5-9 June 2017, Denver, Colorado, 2017.
35. Viet, Rob, Analysis of the flight characteristics of a highly swept cranked flying wing by means of an experimental test, MSc Thesis, Delft University of Technology, 2019.

36. Felder, L.J., Brown, G. V, Hyun Dae, K and Chu, J,. Turboelectric Distributed Propulsion in a Hybrid Wing Body Aircraft, ISABE-2011-1340. 2011.
37. Hyun Dae, K., Felder,L.J., Tong, T.M. and Armstrong, M., Revolutionary Aeropropulsion Concept for Sustainable Aviation: Turboelectric Distributed Propulsion, ISABE-2013-1719, 2013.
38. Liu, Y., X. Sun, V. Sethi, D. Nalianda, Y.-G. Li and L. Wang, Review of modern low emissions combustion technologies for aero gas turbine engines, Progress in Aerospace Sciences, pp. 12-45, 2017.
39. Foust, M.J., D. Thomsen, R. Stickles, C. Cooper and W. Dodds, Development of the GE Aviation Low Emissions TAPS Combustor for Next Generation Aircraft Engines, in 50th AIAA Aerospace Sciences Meeting including the New Horizons Forum and Aerospace Exposition, Nashville, Tennesee, 2012.
40. General Electric (GE), TAPS II Combustor. Continuous Lower Energy, Emissions (CLEEN) Program, Federal Aviation Administration, Washington, DC, 2013.
41. Penanhoat, O. FORUM-AE European Network, in Clean-Sky: European Technology Evaluation workshop, Bruxelles, 2018.
42. Rolt, A.M. and K. G. Kyprianidis, Assesment of New Aeroengine Core Concepts and Technologies in the EU Framework 6 Newac Programme, in 27th International Congress of the Aeronautical Sciences, Nice, 2010.
43. Lee, C.-M., C. T. Chang, J. T. Herbon and S. K. Kramer, NASA Project Develops Next-Generation Low-Emissions Combustor Technologies, in 51st AIAA Aerospace Sciences Meeting including the New Horizons Forum and Aerospace Exposition, Grapevine, Texas, 2013.
44. Tacina, K., D. Podboy, F. Lee and B. Dam, A third-generation swirl-venturi lean direct injection combustor with prefilming pilot injector, in Proceedings of the ASME Turbo Expo 2019: Turbomachinery Technical Conference and Exposition, Phoenix, USA, 2019.
45. Antoine N.E. and I. M. Kroo, Framework for Aircraft Conceptual Design and Environmental Performance Studies, *AIAA Journal,* vol. 43, no. 10, pp. 2100-2109, 2005.
46. Mastropierro, F.S., J. Sebastiampillai, F. Jacob and A. Rolt, Modeling Geared Turbofan and Open Rotor Engine Performance for Year-2050 Long-Range and Short-Range Aircraft, *Journal of Engineering for Gas Turbines and Power,* pp. 041016-1-041016-12, 2020.
47. Suder, K.L., Overview of the NASA Environmentally Responsible Aviation Project's Propulsion Technology Portfolio, in *48th AIAA/ASME/SAE/ASEE Joint Propulsion Conference & Exhibit*, Atlanta, Georgia, 2012.
48. Bass, F.M., A new-product growth model for consumer durables, Management Science 15, 215-227, 1969.
49. Linke, F., Grewe, V., Gollnick, V., The Implications of Intermediate Stop Operations on Aviation Emissions and Climate, Meteorologische Zeitschrift, Jg. 26, Nr. 6, S. 697–709, 2017. doi: 10.1127/metz/2017/0763, 2017.
50. Nuic, A., Poles, D. and Mouillet, V. BADA: An advanced aircraft performance model for present and future ATM systems. Int. J. Adapt. Control Signal Process., 24: 850-866. doi:10.1002/acs.1176, 2010.
51. Deidewig, F., Döpelheuer, A., Lecht, M., Methods to Assess Aircraft Engine Emissions in Flight, in: ICAS Proceedings 1996, Volume 1, ICAS-96-4.1.2, 1996.
